# Hearing It Right, Doing It Safely: A Reflex-Inspired Safety Gatekeeper for Voice-Controlled Exoskeleton Arm Manipulation

**DOI:** 10.3390/biomimetics11090670

**Published:** 2026-09-17

**Authors:** Emanuel Muntean, Monica Leba, Andreea Ionica

**Affiliations:** 1 System Control and Computer Engineering Department, University of Petrosani, 332006 Petrosani, Romania; emanuelmuntean@upet.ro; 2 Management and Industrial Engineering Department, University of Petrosani, 332006 Petrosani, Romania; andreeaionica@upet.ro

**Keywords:** voice control, small language models, intent classification, safety guardrails, assistive robotics, human–robot interaction, exoskeleton robots, wearable robot

## Abstract

Voice interfaces to anthropomorphic robotic arms are typically evaluated without a formal safety layer, or with safety as a post hoc filter trusting the transcript, leaving transcript corruption unaddressed. This paper presents the DAS3 Neuro-Voice Controller, a voice-driven pipeline for a musculoskeletal arm model, evaluated here as a kinematic surrogate for upper-limb exoskeleton and prosthetic control, built on three commitments: on-device intent classification via a 44 M-parameter DistilBERT classifier over a bounded nine-command vocabulary; a deterministic three-layer Gatekeeper enforcing semantic validation, kinematic feasibility, and hard safety constraints independent of classifier confidence; and an upstream Phonetic Interceptor sanitising characteristic ASR mutilations of anatomical and safety terms (“four arm” → “forearm”). Trained on a class-balanced corpus of 3591 expressions and evaluated on fourteen non-native English speakers, the classifier reached 99.95% top-1 accuracy across 1847 oracle-resolvable utterances; the Gatekeeper reduced Priority Override Failure to 0.0%, rejected 100% of out-of-domain speech, and held false-rejection at 2.02%. Against a keyword-spotter baseline the pipeline delivered a 17.4-fold improvement in end-to-end success; the acoustic front-end remains the main weakness. Upstream input repair and downstream deterministic gating emerge as first-class components of safety-critical voice interfaces for assistive systems.

## 1. Introduction

For people living with upper-limb paralysis, amputation, or severe motor impairment, robotic manipulators and exoskeletons promise a route back to independent reaching, grasping, and self-care. Realising that promise depends as much on the control interface as on the mechanism itself: a user who cannot reliably initiate, direct, and above all halt the arm’s motion gains little from its dexterity. Physiological control channels—electromyography, electroencephalography, and inertial measurement—remain the dominant modality in deployed systems, but systematic reviews of upper-limb wearable exoskeletons and exosuits confirm that these signal-based interfaces demand calibration, are sensitive to electrode placement and fatigue, and are frequently unavailable to users with limited residual muscle activity, leaving voice as a secondary channel at best when it is offered at all [1,2].

The past few years have made voice a far more credible primary channel. Large-scale automatic speech recognition (ASR) foundation models such as OpenAI’s Whisper [3] have pushed transcription accuracy to near-human levels across accents and acoustic conditions, and distilled variants bring this capability to offline, edge-deployable hardware. In parallel, large and small language models alike have demonstrated an ability to map free-form natural-language instructions onto structured robot commands, moving assistive interfaces away from rigid keyword grammars and toward genuinely conversational interaction [4,5,6]. Recent work integrating conversational language models with feeding robots and bimanual teleoperation systems has shown that this expressiveness is usable and preferred over constrained alternatives [4,7].

This expressiveness, however, is exactly what makes voice control of an anthropomorphic arm a safety problem rather than a convenience problem. A generic voice assistant that misunderstands a request produces an annoying but harmless error; a language model that emits a spurious motion command for an articulated arm attached to or moving in close proximity to a user’s body converts a linguistic failure into a physical one. Two open technical questions follow from this observation, and the present work argues that both must be resolved together rather than in isolation. The first is architectural: how should safety be enforced so that it cannot be bypassed by an overconfident or adversarial classification, and without depending on a probabilistic component sitting on the critical path between an utterance and an actuator? The second is upstream of the first and is comparatively under-examined: what happens when the transcript itself is wrong? Even State-of-the-Art ASR degrades on non-native, accented, or noisy speech, and degrades further on the low-frequency clinical vocabulary—“glenohumeral”, “supination”, “forearm”—that an anatomically grounded command set requires. A safety layer downstream of a corrupted transcript can only reject it; by the time an error reaches that layer, the information needed to recover the user’s actual intent has already been lost.

This is not merely an engineering convenience; it mirrors a design principle well established in biological motor control. Biological reflex arcs—the withdrawal reflex that pulls a hand from a hot surface, for instance—are routed through the spinal cord rather than the cortex precisely so that a hard-wired, sub-cortical circuit can act before slower, deliberative processing completes. The architecture presented here adopts the same division of labour: the Gatekeeper’s highest-priority tiers intercept STOP and REST commands through direct lexical matching, bypassing the probabilistic classifier entirely, while the classifier itself is reserved for the graded, context-dependent judgements—discriminating one anatomical direction from another—that are better suited to a learned, “cortical” component. This reflex-inspired separation of a fast, deterministic safety pathway from a slower, adaptive interpretive one is what allows the system to remain both expressive and safe under corrupted or ambiguous input.

The closest published systems address one side of this problem well and leave the other largely untouched. VoicePilot and comparable LLM-mediated interfaces for feeding and teleoperation robots [4,7] demonstrate that cloud-hosted, prompt-engineered large language models can absorb considerable paraphrastic variation, but they accept the ASR transcript as given, depend on network connectivity and an external API vendor for latency and availability, and implement safety, where implemented at all, as predefined trajectories or post hoc output filtering rather than as an architecturally independent layer. The emerging safety-guardrail literature takes the complementary view: frameworks such as RoboGuard [8] and modular LLM-as-judge guardrails [9] formalise how a language model’s output should be grounded, filtered, and verified before it reaches an actuator, but both explicitly treat the input side—the fidelity of the transcript the language model receives—as out of scope. No system in either tradition treats upstream transcript repair and downstream deterministic safety enforcement as complementary, first-class architectural components evaluated together.

This paper presents and empirically evaluates the DAS3 Neuro-Voice Controller, a voice-driven control pipeline for an anthropomorphic musculoskeletal arm model that closes this gap through three commitments applied jointly. First, intent classification is performed by a small, fine-tuned, on-device Transformer (a 44 M-parameter DistilBERT classifier with a purpose-built 872-token vocabulary) over a bounded nine-command vocabulary, rather than by a cloud-hosted generative model whose behaviour is not verifiable at the token level. Second, safety enforcement is realised as a deterministic, three-layer Gatekeeper—semantic validation, kinematic feasibility, and hard safety constraints—that gives STOP and REST absolute priority and does not depend on classifier confidence to protect the user. Third, and distinctively, a Phonetic Interceptor sits upstream of both, sanitising the characteristic ASR mutilations of anatomical and safety terms (“four arm” → “forearm”; “cheese” → “cease”) before a corrupted transcript can ever reach the classifier or the Gatekeeper—addressing a failure mode that the guardrail literature has, to date, left unaddressed.

The architecture is evaluated along two complementary axes: a log-derived technical characterisation of the ASR, classification, and safety-gating components across a fourteen-participant live-pipeline cohort, reported against a purpose-built, class-balanced, adversarially stress-tested corpus of 3591 command expressions, and a within-subjects comparison of the resulting interface against keyword-voice and manual-joystick baselines. The remainder of the paper is organised as follows. Section 2 situates this work within the voice-interface, small-language-model, and safety-guardrail studies and identifies the specific architectural gap it addresses. The sections that follow detail the design requirements the architecture must satisfy, the system architecture itself, the construction of the training and evaluation corpora, the empirical evaluation of the resulting pipeline, and a discussion that positions the findings against the closest published systems, before a concluding assessment of what has been established and what remains for future work.

## 2. Related Work

The work presented in this paper sits at the intersection of three converging research streams: voice and natural-language interfaces for assistive manipulation, Transformer-based intent classification with small language models (SLMs), and safety-oriented architectures for language-driven robot control. Rather than surveying each stream exhaustively, the following four subsections identify the specific contributions that motivate our design choices and the architectural gap our system addresses. We deliberately draw on open-access literature, primarily from the last five years, to reflect the rapid maturation of the field since the emergence of large-scale pre-trained Transformer models.

### 2.1. Voice and Natural-Language Interfaces for Assistive Manipulation

Voice control for assistive robotic manipulation has evolved from keyword-driven command grammars toward increasingly flexible natural-language interaction. Early prototypes constrained users to fixed lexicons and simple imperative structures, achieving reliability at the cost of expressive freedom [2]. Systematic reviews of upper-limb wearable exoskeletons and exosuits confirm that most deployed control modalities remain reliant on physiological signals (EMG, EEG, IMU) or on rigid button and joystick interfaces, with voice used as a secondary channel when available [1]. Recent studies have integrated voice input alongside torque-prediction models and adaptive controllers, illustrating the feasibility of multimodal command pipelines but leaving open the question of how spoken language alone can be made reliable enough to serve as a primary channel [5,6].

Voice is best understood as complementary to, rather than a replacement for, these physiological control channels. EMG- and EEG-based intent recognition remain attractive precisely where they excel—continuous, graded control correlated with residual muscle or cortical activity—but they depend on consistent signal quality and are unavailable to users for whom no usable myoelectric or neural signal remains. A voice channel that is itself safety-gated, as described in Section 3.4, does not compete with EMG/EEG pipelines so much as extend assistive arm control to users and situations—fatigue, electrode displacement, or the total absence of a usable physiological signal—in which those channels are degraded or unavailable.

The introduction of large-scale ASR foundation models has fundamentally changed this landscape. OpenAI’s Whisper [3], trained on 680,000 h of multilingual weakly supervised speech, has become the de facto reference architecture for domain-general transcription, with distilled variants specifically designed for edge deployment. Wearable and assistive-robotics work assumes availability of such robust ASR front-ends and shifts the research question upward, toward how to interpret transcribed utterances and safely commit them to actuation [2,5].

Beyond keyword mapping, the past two years have produced a small but influential body of work integrating conversational language models with assistive manipulators. Padmanabha et al. [4] present an LLM-mediated speech interface for a physically assistive feeding robot and articulate the human-centric considerations, including error tolerance and command disambiguation, that separate genuine assistive interfaces from generic voice-to-code pipelines. The gap that remains, and which motivates the present work, is that free-form voice interfaces for anthropomorphic articulated arms are typically evaluated either without a formal safety layer, or with safety implemented as a post hoc filter rather than as an architectural first-class component.

### 2.2. Transformer-Based Intent Classification and Small Language Models

Since the introduction of the Transformer architecture [10], intent classification has migrated from RNN and CNN backbones onto pre-trained encoder families such as BERT and its distilled variants, and onto text-to-text sequence models such as T5 and Flan-T5 [11,12,13]. Comparative work has shown that DistilBERT, despite having far fewer parameters than instruction-tuned LLMs such as LLaMA-3 or Phi-2, achieves competitive or superior accuracy on standard intent-classification benchmarks such as Banking-77 and CLINC-150 while offering substantially lower training and inference cost [14]. A recent review traces the same trajectory in goal-oriented conversational agents and identifies distilled encoder models and compact task-agnostic variants (MobileBERT, edge-optimised attention networks) as the current pragmatic choice for latency-bounded on-device deployment [15].

The label “small language model” (SLM) has emerged as a working category for models in the ~100M–5B parameter range that trade generality for footprint and predictability [4,16]. Their appeal for safety-critical interfaces is threefold. First, on-device inference removes cloud dependency and its associated latency, connectivity, and privacy exposures. Second, a bounded vocabulary and a small classification head over a fixed intent set are inherently easier to certify than an open-ended generative surface. Third, fine-tuning on a modest, purpose-built dataset can yield near-invariant confidence across acoustically diverse users, as demonstrated by recent evaluations of edge-focused intent models [14,15].

In parallel, the T5 family—and in particular Flan-T5, obtained through instruction-tuning at multiple scales [13]—has established itself as a compact sequence-to-sequence baseline for text-to-command mapping, well suited to systems where the desired output is a small structured directive rather than a free-form response. Whether the encoder-only path (DistilBERT) or the encoder–decoder path (Flan-T5) is preferable depends on the granularity of the target vocabulary and the tolerance for out-of-distribution utterances; both are represented in the current landscape [11,13].

### 2.3. LLM and SLM Integration for Robot Control

The past five years have produced a rapid succession of frameworks integrating large language models with robotic manipulators. Foundational works such as SayCan [17] and Code-as-Policies [18] established the pattern of using an LLM to translate natural-language goals into either skill sequences or executable code, subsequently grounded in the robot’s affordances. Zhang et al. [19] survey this trajectory and highlight the tension between the expressive power of LLM-driven interfaces and the safety and reproducibility requirements of physical robotic control. A more recent review by Liu et al. [16] catalogues the state of LLM integration across locomotion, navigation, manipulation, and voice-based interaction, and notes that safety enforcement—as distinct from mere planning accuracy—remains under-treated in the literature.

For anthropomorphic arm control specifically, Fei et al. [7] present a bimanual teleoperation framework in which an LLM interprets voice instructions to select from a bounded set of manipulation skills, reducing operator workload while retaining human control of the primary arm. Their evaluation confirms that natural-language mediation of an articulated effector is feasible when the command surface is constrained to a small, well-defined skill vocabulary, and that the interaction quality is bounded by the reliability of the language-understanding front-end rather than by the underlying kinematics.

A common thread across this body of work is reliance on cloud-hosted general-purpose LLMs (GPT-3.5, GPT-4, LLaMA-2/3) whose behaviour is not verifiable at the token level and whose failure modes include semantic hallucination, misinterpretation of compound commands, and adversarial vulnerability. In the assistive setting, these failure modes are not merely inconvenient: an LLM that emits a spurious motion command for an anthropomorphic arm attached to or moving in proximity to a user is a physical-safety hazard, not a linguistic error [16,19]. This concern has recently driven work toward SLM-based, on-device pipelines with bounded output surfaces [4,14,16], and toward explicit safety layers, discussed next.

### 2.4. Safety Layers and Guardrails for Language-Driven Robot Control

The recognition that LLM/SLM outputs cannot be trusted unconditionally has crystallised into an emerging sub-literature on safety guardrails for language-enabled robots. Ravichandran et al. [8] propose RoboGuard, a two-stage guardrail architecture in which a shielded root-of-trust language model first grounds high-level safety rules into context-specific specifications (such as temporal-logic constraints), and a second stage enforces those specifications at execution time. The authors formalise four desiderata—utility preservation, efficiency, contextual grounding, and adversarial robustness—that have become a reference for subsequent work on safety wrappers around LLM-driven robots.

Modular safety-guardrail proposals have since extended this line of thinking with monitoring layers that combine LLM-as-judge validation, conformal prediction for uncertainty, constitutional-rule verification, and physical trajectory projection onto safe sets [9]. The consistent conclusion of this literature is that decision-level filtering alone cannot guarantee physical safety: adversarial or hallucinated outputs can slip through content-only filters, and safety must therefore be enforced at multiple architectural layers with mutually redundant mechanisms.

A recurring blind spot across these frameworks, however, is that they operate downstream of the language model—filtering, ranking, or clamping its outputs—while treating the input side as a solved problem. In voice-driven systems this assumption is precisely where reliability breaks down. Even State-of-the-Art ASR degrades on accented or noisy speech, producing transcriptions in which anatomical or safety terms are systematically mutilated (“forearm” → “four arm”, “cease” → “cheese”, “abort” → “a board”). When such corrupted text reaches a downstream language model, downstream safety layers can only reject, never repair. To our knowledge, no prior open-access study has integrated an upstream phonetic-repair stage with a downstream deterministic safety hierarchy as complementary, first-class architectural components.

### 2.5. Positioning of the Present Work

The system evaluated in this paper differs from prior work along three axes simultaneously. First, intent classification is performed by an on-device SLM with a bounded vocabulary rather than by a cloud LLM, aligning with the direction indicated by recent edge-focused evaluations [4,14,16]. Second, safety enforcement is realised through a deterministic, tiered Gatekeeper that pre-empts probabilistic classification for emergency commands, in the spirit of but distinct from downstream guardrail wrappers [8,9]. Third, an upstream phonetic-repair layer explicitly addresses ASR-induced input corruption, an issue that the safety-guardrail literature has thus far treated as out of scope. Together these three commitments constitute the architectural contribution empirically evaluated in the sections that follow.

## 3. Materials and Methods

### 3.1. Architecture Overview

**Figure 1:**
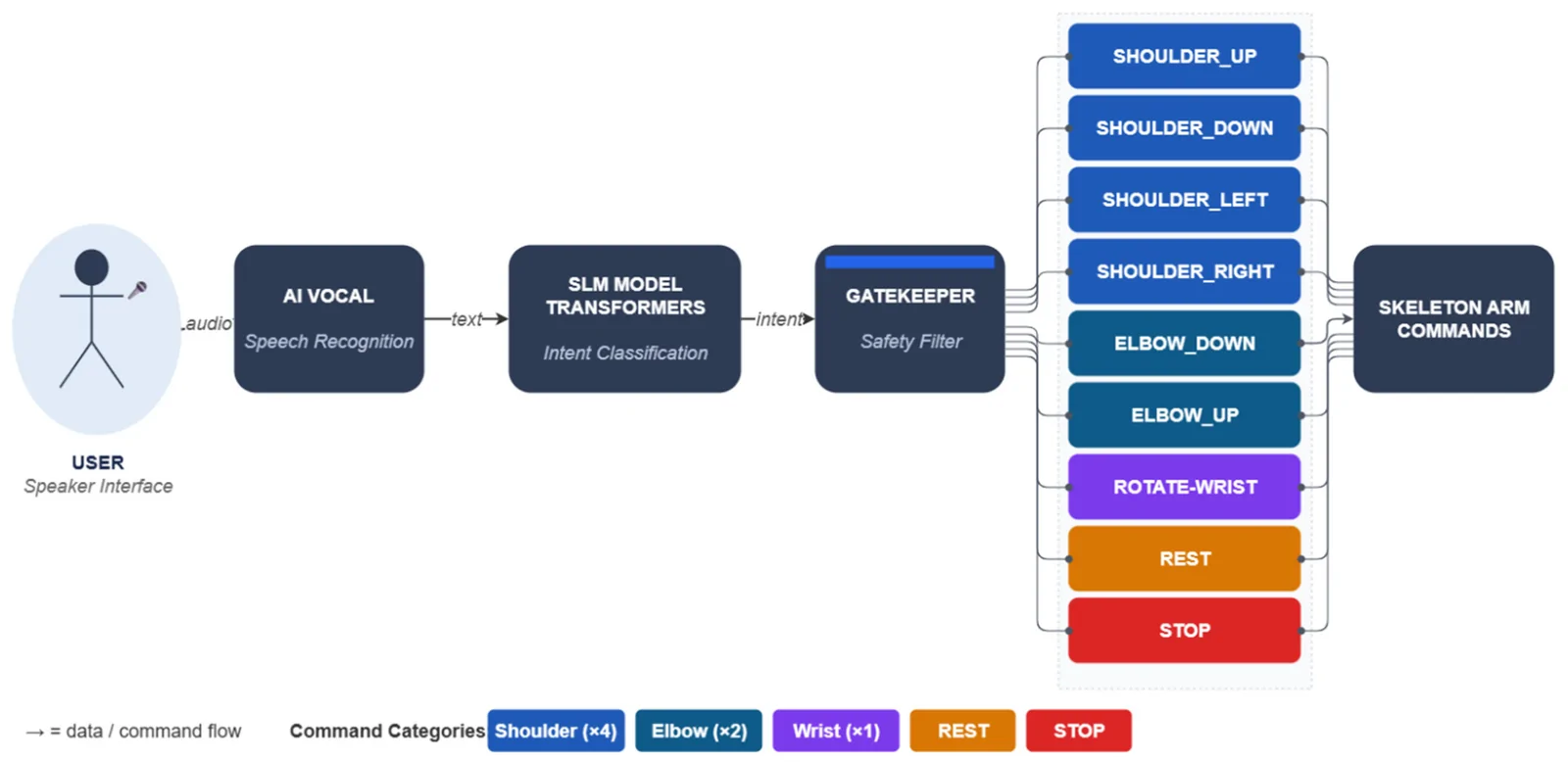
End-to-end block diagram of the DAS3 Neuro-Voice Controller.

The DAS3 Neuro-Voice Controller is a serial, left-to-right pipeline that progressively transforms a spoken utterance into a discrete motor command dispatched to the musculoskeletal model (Figure 1). A human operator issues a vocal instruction captured by a near-field microphone and forwarded to the AI Vocal module, which returns a text transcription with confidence score. The transcript is routed to the SLM Transformer, a fine-tuned encoder-only classifier that maps the utterance onto one of nine command labels. Before any actuation, the classified intent traverses the Gatekeeper—the primary safety enforcement layer—and, if all checks succeed, is dispatched to the Skeleton Arm Commands execution layer, which translates the label into a kinematic directive within the OpenSim environment. So, voice input is transcribed by the AI Vocal module, classified by the SLM Transformer, validated by the Gatekeeper, and dispatched as one of nine atomic commands (four shoulder directions, two elbow directions, wrist rotation, REST, STOP) to the Skeleton Arm Commands execution layer.

### 3.2. Voice Front-End (AI Vocal)

The ASR front-end is implemented using Distil-Whisper v3.5 (distil-whisper/distil-small.en), a 166 M-parameter distilled variant of Whisper that delivers approximately six-fold inference acceleration at within one percentage point of the reference WER. The model executes fully offline, eliminating network-induced latency, and is constrained to English input regardless of the participant’s native language. Audio capture is managed via the speech_recognition v3.1.3 Python library with a hard static noise gate set to three times the ambient baseline (floor 1000 energy units), restricting valid capture to utterances within roughly 100 cm of the transducer. End-of-utterance detection uses a 0.3 s pause_threshold and 0.2 s non_speaking_duration. Inference is offloaded to a single-worker ThreadPoolExecutor and runs in FP16 when CUDA is available. A Phonetic Interceptor post-processes the raw transcript to sanitise characteristic ASR mutilations of anatomical and safety terms (“four arm” → “forearm”; “herbo” → “elbow”; “cheese” → “cease”) before the text reaches the Gatekeeper.

Because the command vocabulary is restricted to a closed set of nine labels, traditional Word Error Rate is not the primary quality metric. Recognition performance is instead quantified as the Vocal AI First-Pass Recognition Rate (FPRR)—the proportion of voice inputs that cleared the Gatekeeper on the initial attempt. Across the fourteen-participant cohort the mean FPRR was 76.73% (SD = 10.76%, range 60.43–94.74%). The strong inverse correlation between FPRR and Gatekeeper Block Rate (r = −0.998, *p* < 0.0001) confirms that ASR first-pass fidelity is the dominant determinant of downstream pipeline behaviour.

### 3.3. SLM Transformer Module

#### 3.3.1. Base Model and Tokenizer

The intent classifier is a custom fine-tuned DistilBERT sequence classifier, initialised from distilbert-base-uncased via the Hugging Face Transformers v5.17.0 library. By binding the model to a purpose-built anatomical vocabulary, the network is instantiated at exactly 44,189,193 trainable parameters—substantially smaller than BERT-base (110 M) while retaining the bidirectional self-attention of the encoder-only family. DistilBERT was selected over generative alternatives (e.g., T5, GPT-2) on three converging grounds. First, its encoder-only design is architecturally optimal for fixed-class intent classification, where the task demands a single categorical label rather than an open-ended sequence generation. Second, distillation reduces both memory footprint and inference latency by approximately 40% relative to BERT-base while retaining over 97% of its representational capacity—a trade-off critical for real-time deployment. Third, the Hugging Face AutoModelForSequenceClassification API v3.0.2 enables seamless integration of the classification head with the custom tokenizer and the downstream Gatekeeper decision logic.

A custom WordPiece tokenizer was trained from scratch, inheriting the structural rules and special tokens ([CLS], [SEP]) of the distilbert-base-uncased blueprint but re-deriving the vocabulary exclusively from the 2872 training phrases with a hard ceiling of 1500 tokens. The resulting vocabulary comprises exactly 872 domain-specific tokens, encoding the complete anatomical and command lexicon with near-zero redundancy and drastically reduced input tensor dimensionality.

#### 3.3.2. Dataset, Class Balancing, and Fine-Tuning

Nine text corpora—one per command class—were compiled through a hybrid human + LLM-assisted expansion pipeline (Section 4.1), aggregated, and cross-file deduplicated. All classes were downsampled to a balanced 399 samples per class, yielding 3591 expressions. An 80/20 stratified split (random_state = 42) produced 2872 training and 719 validation samples with identical class proportions across partitions. Given the safety-critical nature of emergency commands, a hierarchical Signal-Booster augmentation was applied exclusively to the training partition: STOP received a ×15 multiplier, REST an ×8 multiplier, and all seven anatomical movement commands a ×2 multiplier, expanding the training pool from 2872 to 11,818 weighted samples and biassing the dense layers toward safety-critical intents under ambiguous acoustic conditions. This asymmetric weighting is a deliberate safety-oriented design choice rather than an unexamined side effect, and it carries a corresponding trade-off worth stating explicitly. Biassing the classifier toward STOP and REST increases the risk that an acoustically ambiguous or noisy movement utterance is misclassified as a cessation command, interrupting a motion the participant did not intend to stop; the blind-test confusion analysis (Section 3.5) reports the reverse confusion direction as the dominant one observed (STOP misclassified as REST in 40 of 200 samples, both cessation-semantic classes), with no movement-to-STOP confusion among the boundaries characterised there, though a full nine-class confusion breakdown was not exhaustively reported. In live operation, this exposure is further narrowed because STOP and REST utterances that match a Gatekeeper Tier 1/2 keyword pattern bypass the SLM entirely (Section 3.4); the boosted decision boundary is only reachable when an utterance contains no such literal trigger and falls through to SLM inference. We consider a false-positive STOP—an unwanted but harmless interruption—the substantially preferable failure mode to a false-negative STOP under safety-critical operation, consistent with standard fail-safe design practice, and accept the resulting cost to user comfort (an occasional unnecessary pause) as the deliberate consequence of that priority. We have not directly measured either the frequency of spurious SLM-mediated STOP activation under noisy acoustic conditions or its subjective comfort cost to participants, and we identify both as concrete targets for future evaluation. Fine-tuning was executed via the Hugging Face Trainer API with a learning rate of 5 × 10^−5^, weight decay 0.05, and 30 epochs, with end-of-epoch validation.

#### 3.3.3. Output Schema and Inference Budget

At inference, the SLM applies a Softmax function over the raw output logits to produce explicit probability estimates across the nine-class label space. The resulting structured decision object exposes four typed fields (Table 1) that constitute the complete command specification passed to the downstream Gatekeeper. Every field is typed and bounded; the downstream Gatekeeper (Section 3.4) refuses to forward any decision object whose fields fail the schema well-formedness check.

On the evaluation hardware, the mean SLM inference latency is 21.69 ms per utterance—well within the 2.5 s end-to-end pipeline budget—and the blind offline accuracy reaches 96.42% on a 1760-expression test set (Section 4). In live operation, a substantial proportion of STOP and REST events bypass the SLM entirely through deterministic lexical matching at Gatekeeper Tiers 1 and 2, dispatching at effectively zero classifier latency. The memory footprint of the combined tokenizer and fine-tuned classifier is compact enough for deployment on mid-range laptop hardware without discrete GPU acceleration; deployment on constrained edge platforms such as the NVIDIA Jetson Nano is feasible given the 44 M parameter count, though platform-specific benchmarking remains a direction for future work.

### 3.4. Gatekeeper Module

**Figure 2:**
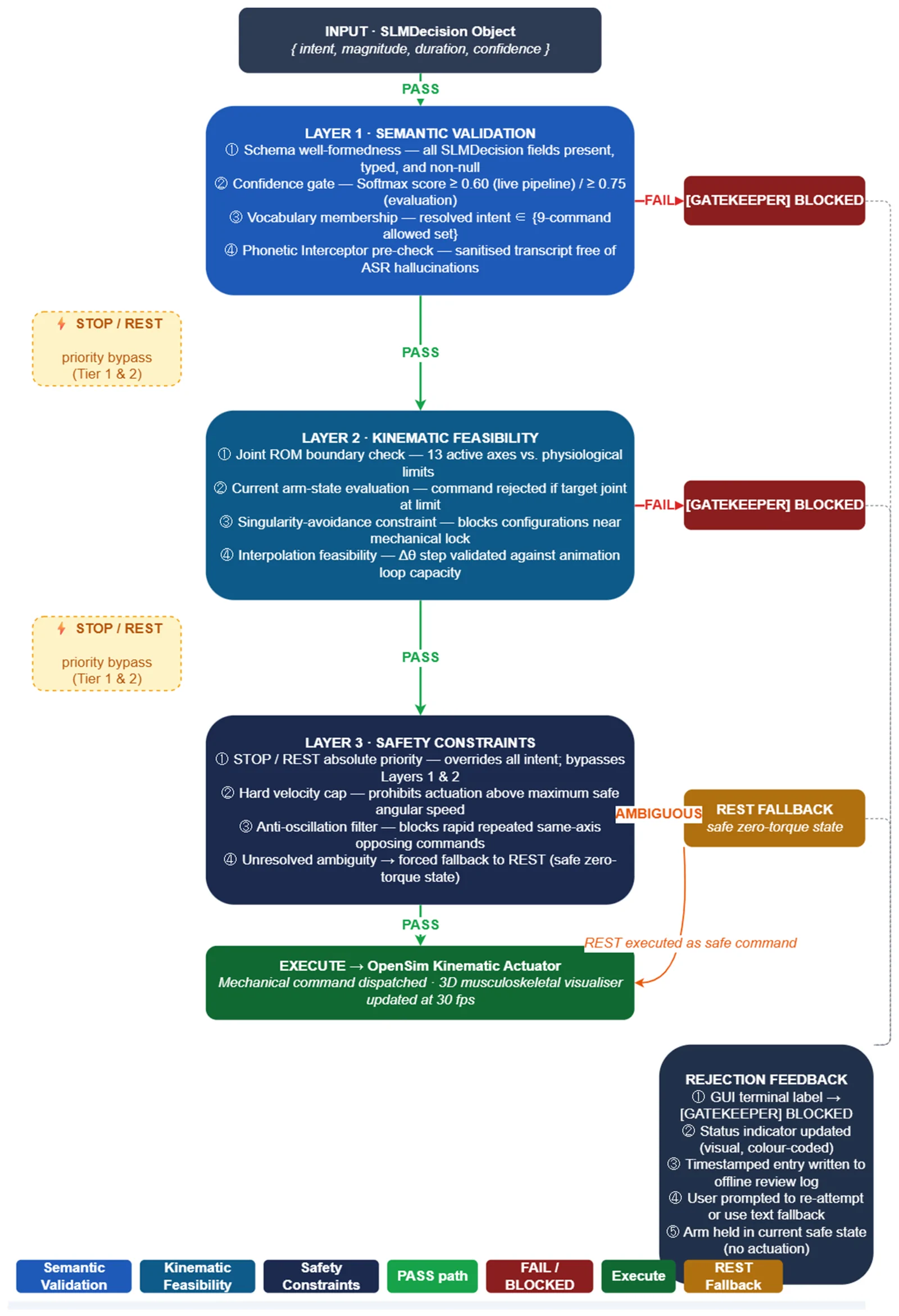
Three-layer Gatekeeper architecture.

The Gatekeeper is the primary safety and validation contribution of the system. Positioned between the probabilistic SLM output and the deterministic OpenSim actuation, it implements a three-layer sequential decision architecture (Figure 2) that transforms a raw decision object into either a verified command or a structured rejection. Unlike the upstream neural components, the Gatekeeper enforces hard, rule-based invariants that cannot be overridden by classifier confidence alone. The three-layer architecture described here is the outcome of a V1→V2 design evolution. Version 1 (V1) implemented the Gatekeeper as a flat, single-pass lexical filter—the PRIORITY_WORDS algorithm—in which the STOP/REST safety check and the two-keyword anatomical admission check operated in parallel at the same decision level, without a fixed order of precedence. A compound utterance satisfying both conditions (e.g., “stop moving the shoulder up”) could therefore clear the gate and be promoted to the SLM, whose attention weights, biassed toward the “shoulder” + “up” co-occurrence, resolved the intent to SHOULDER_UP rather than to STOP—the Priority Override Failure. Version 2 (V2) replaces this flat gate with the strict tiered hierarchy detailed below: STOP (Tier 1) and REST (Tier 2) are evaluated first and short-circuit the pipeline before the SLM is invoked, structurally removing the possibility that any combination of anatomical words overrides a safety intent.

Layer 1—Semantic Validation runs four sub-checks: schema well-formedness (intent, magnitude, duration, confidence present and correctly typed), a confidence gate at 0.60 live/0.75 offline, vocabulary-membership verification against the nine-command allowed set, and a Phonetic Interceptor pre-check confirming that upstream sanitization has resolved known ASR mutilations.

Layer 2—Kinematic Feasibility validates that the requested motion is physically executable. A range-of-motion check against the thirteen-axis physiological limit table (spanning the SC, AC, GH, EL, PS, and WR complexes; see Section 3.6) rejects any command whose target would exceed its bounds—for example, SHOULDER_UP is blocked when GH_z is already at 84°. A current arm-state check prevents silent no-ops, a singularity-avoidance constraint flags Glenohumeral configurations approaching co-planarity, and an interpolation-feasibility check verifies compatibility with the 30 fps smoothing loop.

Layer 3—Safety Constraints enforces system-level policies with higher priority than the intent classifier output. STOP and REST are matched deterministically against dedicated keyword arrays and dispatched with absolute priority, bypassing Layers 1 and 2 entirely, regardless of any co-occurring anatomical vocabulary. This architectural bypass eradicated the Priority Override Failure observed in the V1 pipeline—where “stop moving the shoulder up” was misclassified as SHOULDER_UP—reducing the priority failure rate to 0.0% in live testing. A hard velocity cap prohibits actuation above the maximum safe angular speed regardless of the commanded magnitude field; an anti-oscillation filter detects and blocks sequences of rapid, same-axis opposing commands indicative of user hesitation or acoustic noise; and any command that clears all preceding checks but cannot be resolved to a deterministic mechanical intent is subject to a mandatory REST fallback, transitioning the arm to the safe zero-torque resting state rather than attempting a best-guess execution of an ambiguous directive.

On rejection at any layer, the system triggers five concurrent feedback mechanisms: the GUI terminal label is updated with the “[GATEKEEPER] BLOCKED” status string, providing immediate visual confirmation of the rejection event; the colour-coded status indicator in the Tkinter controller panel is refreshed to the blocked state, offering a modality-independent visual cue for users monitoring the interface at a distance; a timestamped rejection entry—including the raw transcription, the SLM output, the rejection layer, and the specific sub-check that triggered the block—is written to the offline review log for subsequent performance analysis; the user is implicitly prompted to re-attempt the utterance via the voice channel or escalate to the manual text fallback pathway, which bypasses the acoustic pipeline entirely; and the robotic arm is held in its current safe state with no actuation pending successful command resolution, ensuring that the physical manipulator never moves in response to a rejected input.

The reflex analogy introduced in Section 1 corresponds to specific structural and functional properties of the Gatekeeper rather than serving only as a descriptive metaphor. Structurally, a biological reflex arc comprises a receptor, an afferent pathway, a low-order integration centre, an efferent pathway, and an effector; Tier 1/2 of the Gatekeeper occupies the role of the integration centre, receiving its input from the acoustic afferent pathway (near-field microphone, ASR, Phonetic Interceptor) and dispatching directly to the effector (the Skeleton Arm Commands execution layer) without routing through the SLM—the architectural analogue of the “cortical” component. Functionally, the correspondence is sharpest in the override behaviour that motivated the V1 → V2 redesign: a defining property of biological reflexes is that a fast, low-order circuit can interrupt an ongoing higher-order motor plan, most clearly documented in reciprocal-inhibition circuits that suppress an antagonist voluntary command when a withdrawal reflex fires. The V1 Priority Override Failure—in which “stop moving the shoulder up” was resolved by the SLM’s attention weights to SHOULDER_UP rather than STOP—was precisely a failure of this override property: the fast pathway had no structural priority over the slow one. The V2 tiered hierarchy restores it by construction, giving Tier 1/2 absolute precedence regardless of concurrent SLM-bound vocabulary, which is what eliminated the failure mode (Section 5.6). The one dimension along which we cannot yet substantiate the analogy quantitatively is latency: while the Tier 1/2 bypass avoids the SLM’s 21.69 ms mean inference pass by construction, the present dataset records a single session-level end-to-end aggregate (Section 5.7) rather than per-component timestamps, so a reflex-pathway-versus-cortical-pathway latency differential—the property that gives biological reflexes their speed advantage—cannot presently be reported with the same granularity as an EMG-measured reflex latency. Per-tier timestamp logging is identified as a priority instrumentation addition for this purpose.

### 3.5. The Nine-Command Vocabulary

The nine-command vocabulary reflects a deliberate trade-off between the expressive completeness required for functional upper-limb assistive control and the classification reliability sustainable by a 44 M-parameter classifier trained on 399 samples per class. Anatomically, four Glenohumeral directions (SHOULDER_UP/DOWN on GH_z, SHOULDER_LEFT/RIGHT on GH_y) cover reaching in the sagittal and frontal planes; two elbow directions (ELBOW_UP/DOWN on EL_x) provide the primary distal degree of freedom for reach-and-grasp; ROTATE_WRIST (PS_y) adds the rotational degree of freedom essential for tool-orientation Activities of Daily Living; and REST and STOP are non-negotiable safety primitives required by ISO 10218 [20] collaborative-robot guidelines. Table 2 provides the complete mapping from each command label to its physical kinematic effect, as implemented in the execution layer. Each movement command triggers an INCREMENT schema token that adjusts the target coordinate array q_tgt by the fixed angular step along the designated joint axis, followed by 30 fps interpolation to the new target. REST issues a STATE token resetting all axes to the zero-pose; STOP issues an EMERGENCY token that freezes actuation with zero computation latency.

Empirically, the nine-class configuration reaches the operational ceiling of the current training regime. The overall 96.42% blind accuracy conceals two structural confusion boundaries: STOP achieves F1 = 0.89 with 40 of its 200 evaluation samples misclassified as REST (both being cessation semantics whose lexical overlap directly competes for the classifier’s attention weights), and ELBOW_UP achieves 88% recall with 20 samples confused with ELBOW_DOWN (a directional minimal pair whose only discriminating signal is the antonym “up” vs. “down” or “in” vs. “out”). Both boundaries indicate that the classifier is at the edge of its reliable semantic discrimination capacity under the current training scale. Adding further commands—particularly semantically adjacent variants such as WRIST_LEFT/RIGHT or a dedicated FOREARM_EXTEND—would predictably degrade accuracy below the 95% threshold clinically accepted for assistive-device reliability without a proportional expansion of the training corpus.

### 3.6. Skeleton Arm Commands Execution Layer

**Figure 3:**
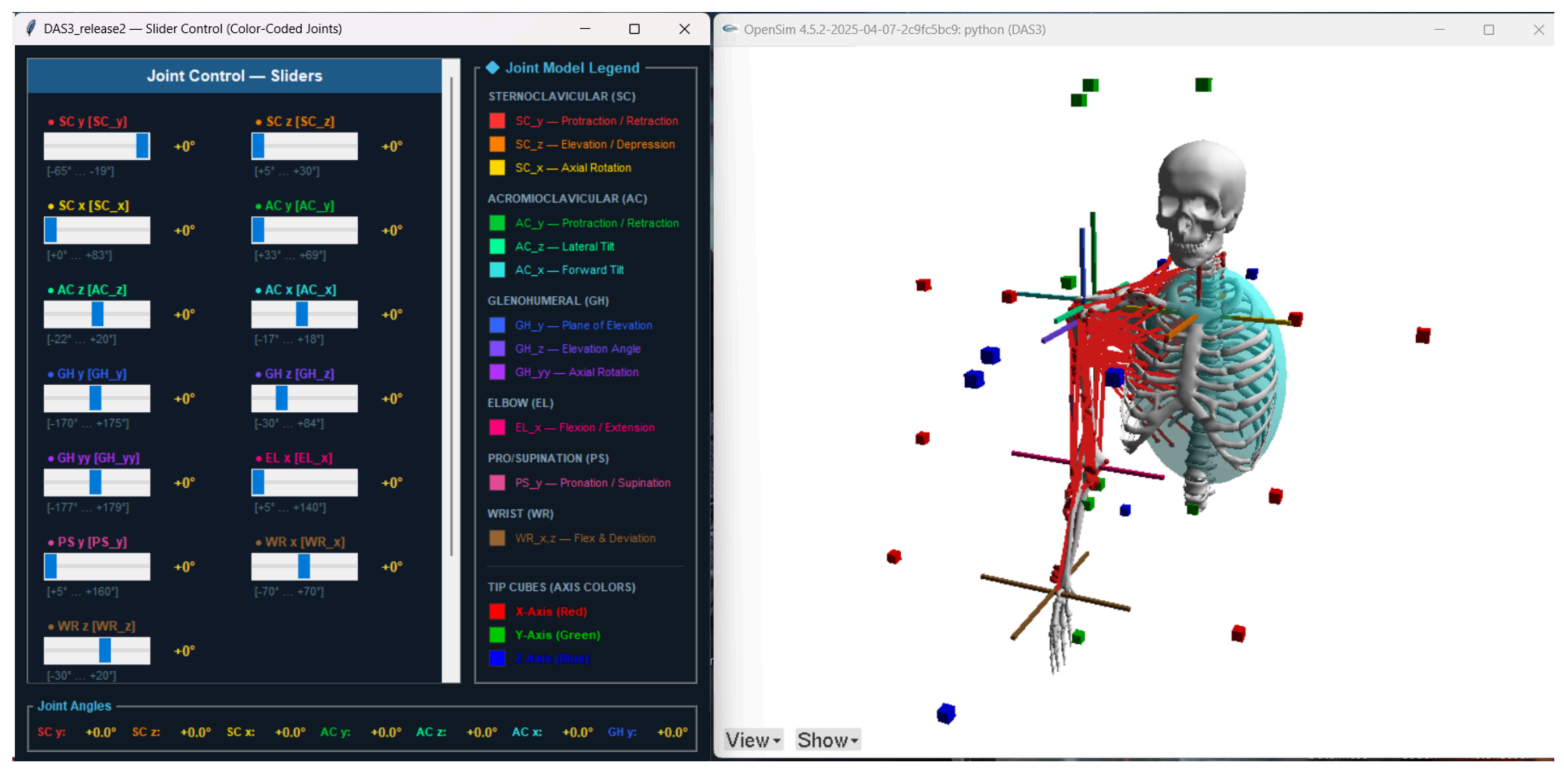
OpenSim DAS3 execution environment.

The execution layer receives the verified decision object from the Gatekeeper and translates its schema token into a kinematic directive within the OpenSim musculoskeletal environment (Figure 3). This translation is intentionally thin: it maps the three schema types—INCREMENT, STATE, EMERGENCY—onto coordinate-array manipulations, insulating the upstream natural-language pipeline from any hardware-specific detail. The execution environment is built upon the OpenSim v4.6 Python API (osim.Model), which interfaces directly with the Dynamic Arm Simulator 3 (DAS3) musculoskeletal model of the right upper limb. At initialization, a dynamic state-check routine parses the model’s full coordinate set and programmatically unlocks all target joints across six anatomical complexes—Sternoclavicular (SC_), Acromioclavicular (AC_), Glenohumeral (GH_), Elbow (EL_), Pro/Supination (PS_), and the additionally integrated Wrist (WR_)—via setLocked(state, False), expanding the active degrees of freedom beyond the default DAS3 configuration to enable complete wrist articulation alongside the shoulder and elbow complexes. The Tkinter dashboard (left) provides per-joint sliders colour-coded to match the axis colours of the 3D visualizer (right), together with a status label that mirrors every Gatekeeper decision—successful execution, blocked rejection, or safety override—in real time.

On INCREMENT, the target coordinate array q_tgt is adjusted for the designated axis by the fixed angular step (e.g., GH_z step = +20° on SHOULDER_UP), and a background kinematic thread—isolated from the Tkinter UI loop—drives the model from its current joint state to the new target at 30 fps under step = sign(err) × min(|err|, speed × Δt), producing continuous physiologically plausible trajectories. STATE resets all unlocked coordinates to the zero-pose; EMERGENCY bypasses the interpolator entirely, freezing q_tgt with zero latency. All angular displacements are bounded by the physiological range-of-motion limits established in Table 3 and enforced by the Gatekeeper’s Layer 2 kinematic feasibility check, ensuring that no voice command can drive any joint axis beyond its anatomically safe range regardless of repeated application.

The real-time control dashboard, implemented in Tkinter, provides bidirectional visual feedback throughout execution. Per-joint sliders are colour-coded to match the exact hexadecimal axis colours rendered in the 3D OpenSim visualiser, creating a spatially coherent mapping between the physical 3D model and the numerical readout panels. A dedicated “Action: [COMMAND]” status label, mirrored across the main terminal window and the compact controller view, updates synchronously with every Gatekeeper decision—a successful execution, a “[GATEKEEPER] BLOCKED” rejection, or a safety override event—providing the operator with an unambiguous, latency-free confirmation of the pipeline state at all times.

A central architectural claim of this work is that the natural-language pipeline in Section 3.1, Section 3.2, Section 3.3, Section 3.4 and Section 3.5 is hardware-agnostic by design. The Gatekeeper’s output carries a validated intent label, an angular magnitude, an interpolation duration, and a schema type—a self-contained command specification containing no reference to any specific actuator, communication protocol, or simulator. The execution layer described here is therefore one instantiation of a general command consumer: the identical stream can, without upstream modification, drive a physical upper-limb exoskeleton via a motor-controller API that maps INCREMENT(axis, direction, Δθ) to servo or pneumatic actuator commands on the corresponding joint; a myoelectric or body-powered prosthetic arm, where INCREMENT maps to grip aperture or terminal-device rotation commands indexed against the prosthetic’s available DoF set; or a simulated avatar in any physics engine that exposes a joint-angle API (Unity, Gazebo, MuJoCo) by replacing only the q_tgt update function with the target engine’s coordinate setter. This decoupling is structurally guaranteed by the strict schema abstraction enforced at Gatekeeper Layer 1: because no command is permitted to reach the execution layer unless normalised to one of three schema tokens with fully typed fields, the downstream actuator interface is never exposed to raw linguistic output, idiosyncratic model confidence values, or ASR transcription artefacts. The pipeline thus functions as a universal voice-to-motion translator whose natural-language intelligence is decoupled from its physical substrate.

## 4. Dataset Construction

This section documents the natural language corpus underpinning the SLM classifier of the DAS3 Neuro-Voice Controller. The dataset design reflects three concurrent objectives: paraphrastic diversity within each command class, strict class balance to prevent frequency-driven attention artefacts, and structured adversarial coverage to stress-test the Gatekeeper’s safety-tier enforcement.

### 4.1. Vocabulary and Elicitation

The command vocabulary comprises the nine atomic intents defined in Section 3.5: four Glenohumeral directions (SHOULDER_UP, SHOULDER_DOWN, SHOULDER_LEFT, SHOULDER_RIGHT), two elbow directions (ELBOW_UP, ELBOW_DOWN), one forearm-rotation intent (ROTATE_WRIST), and the two safety primitives (REST, STOP). Corpus construction followed a four-stage protocol designed to yield paraphrastic diversity while preserving intent fidelity.

Stage 1—seed generation: for each of the nine intents, a set of prototype utterances was manually authored by the research team to span four discourse registers—formal (“please raise the shoulder”), informal (“shoulder up”), polite-request (“could you lift my shoulder”), and terse-imperative (“lift”). The seed set deliberately included the anatomical, positional, and action-verb variants that a naïve user might produce when addressing a robotic arm for the first time. Stage 2—hybrid human + LLM-assisted expansion: each seed was expanded through a paraphrase-generation pass in which a large language model was prompted to produce syntactic and lexical variants preserving the intent label, followed by manual review of every candidate; this hybrid procedure combined the coverage breadth of automated generation with the intent-fidelity control of human curation and produced approximately 400 unique expressions per class (~3600 total). Stage 3—curation: all candidate utterances were reviewed by the research team a second time to eliminate command-intent drift, off-vocabulary constructions, and near-duplicate phrasings that survived the LLM expansion. Stage 4—balancing: the curated corpus was clamped at exactly 399 expressions per class through stratified downsampling (Section 4.2).

### 4.2. Dataset Composition

#### 4.2.1. Corpus Balance and Signal-Booster Augmentation

A global deduplication sweep identified and purged one cross-file leakage instance (the phrase “lift the forearm higher”, duplicated across two corpora). All nine command categories were then clamped to exactly 399 samples per class, producing a balanced master corpus of 3591 expressions. This balancing step was specifically adopted to prevent the classifier’s attention heads from exploiting differential class frequency—a well-documented failure mode in intent classification systems with naturally unequal vocabulary richness. A stratified 80/20 split (random_state = 42) then yielded 2872 training samples and 719 validation samples, with identical class proportions preserved across both partitions.

Prior to fine-tuning, the training partition was processed by the Signal-Booster augmentation pipeline, which applied a hierarchical duplication multiplier reflecting the safety-critical priority structure of the command vocabulary: STOP expressions received a ×15 multiplier, REST expressions a ×8 multiplier, and all seven anatomical movement commands a ×2 multiplier—expanding the effective training pool from 2872 to 11,818 weighted samples. This intentional distributional skew biases the model’s dense classification layers toward safety-override intents under acoustically ambiguous conditions, directly mitigating the Priority Override Failure vulnerability identified during V1 Gatekeeper testing.

#### 4.2.2. Train/Validation/Test Splits and Disjointness

Three strictly separated data partitions are maintained throughout the experimental protocol, summarised in Table 4. The training partition (2872 pre-boost samples → 11,818 boosted) and validation partition (719 samples) are drawn from the same 3591-expression balanced corpus via stratified split and are used exclusively during SLM fine-tuning and per-epoch convergence monitoring, respectively. The blind test set constitutes an entirely independent evaluation corpus of 1760 unique unmultiplied expressions constructed through a separate generation pipeline with no expression-level overlap with the training or validation partitions. It preserves a near-uniform per-class distribution—195 expressions per class for eight command categories and 200 expressions for STOP, whose additional test density was specifically included to characterise the STOP↔REST confusion boundary more precisely. The blind test set was evaluated under the same 0.75 confidence threshold operative during offline evaluation, and no Signal-Booster weighting was applied, ensuring that the reported 96.42% accuracy reflects genuine generalisation performance rather than frequency-inflated recall.

Because both the training corpus and the blind test set are text-only datasets, speaker-level disjointness in the acoustic sense is not applicable; lexical disjointness—the absence of identical expression strings across partitions—was enforced by the global deduplication sweep. For the live voice evaluation cohort (Section 5.1), all fourteen participants contributed exclusively to the evaluation phase; no participant’s voice recordings were used in ASR training, as the Distil-Whisper front-end was deployed pre-trained without speaker-specific fine-tuning.

#### 4.2.3. Adversarial Utterance Subset

Beyond the standard nine-class blind test, a structured adversarial subset was assembled to stress-test the integrated pipeline—specifically the Gatekeeper’s lexical enforcement tiers and the SLM’s priority discrimination capacity under edge-case conditions. The subset is organised into five functionally distinct categories (Table 5). Category 1 (Canonical Valid Commands) establishes the Gatekeeper’s positive-control baseline: complete, well-formed anatomical commands that should be accepted and correctly classified by the SLM, forming the mirror case of Category 3’s rejections. Category 2 (Paraphrased/Synonym Commands) verifies Layer 1 vocabulary tolerance and the SLM’s generalisation to semantically equivalent phrasings of valid commands, confirming that paraphrases and synonym substitutions still resolve to the intended command label. Category 3 (Partial/Truncated Commands) contains valid vocabulary words that fail to meet the Gatekeeper’s minimum anatomical-keyword threshold and should be blocked at Tier 5 without ever reaching the SLM. Category 4 (Out-of-Domain Chatter) simulates ambient conversation unrelated to the command vocabulary and tests the false-positive activation rate. Category 5 (Conflicting Compound Commands) directly targets the Priority Override Failure vulnerability observed in the V1 Gatekeeper: utterances containing both a safety-tier keyword (from the STOP or REST arrays) and one or more anatomical movement verbs in the same sentence. Under the V2 Hierarchical Gatekeeper, Tier 1 and Tier 2 intercept and classify these expressions before the SLM is invoked, reducing the Priority Override Failure rate to exactly 0.0 % across the entire Category 5 subset.

### 4.3. Annotation and Inter-Rater Reliability

The raw corpus, evaluated prior to clamping, contains 3601 linguistic expressions, each independently labelled with a robot-arm command by two raters. Both raters chose from the same set of nine categories: ELBOW_DOWN, ELBOW_UP, REST, ROTATE_WRIST, SHOULDER_DOWN, SHOULDER_LEFT, SHOULDER_RIGHT, SHOULDER_UP, STOP.

Agreement between the two raters was quantified using Cohen’s kappa coefficient (*κ*), which measures inter-rater agreement while correcting for the level of agreement expected by chance:

$$\kappa =\frac{P_{o}-P_{e}}{1-P_{e}}$$

where *P_o_* is the observed proportion of exact agreement between the two raters, and *P_e_* is the proportion of agreement expected if the two raters assigned labels independently, based on each rater’s marginal label frequencies (Table 6).

Cohen’s kappa was *κ* = 0.9850 (95% CI: 0.9808–0.9892), computed from an observed agreement of 98.67% against a chance-expected agreement of 11.11%. Following the Landis and Koch [21] benchmark scale, this value corresponds to almost perfect agreement. The result is highly significant (z = 458.6), indicating the agreement is far greater than would occur by chance.

**Figure 4:**
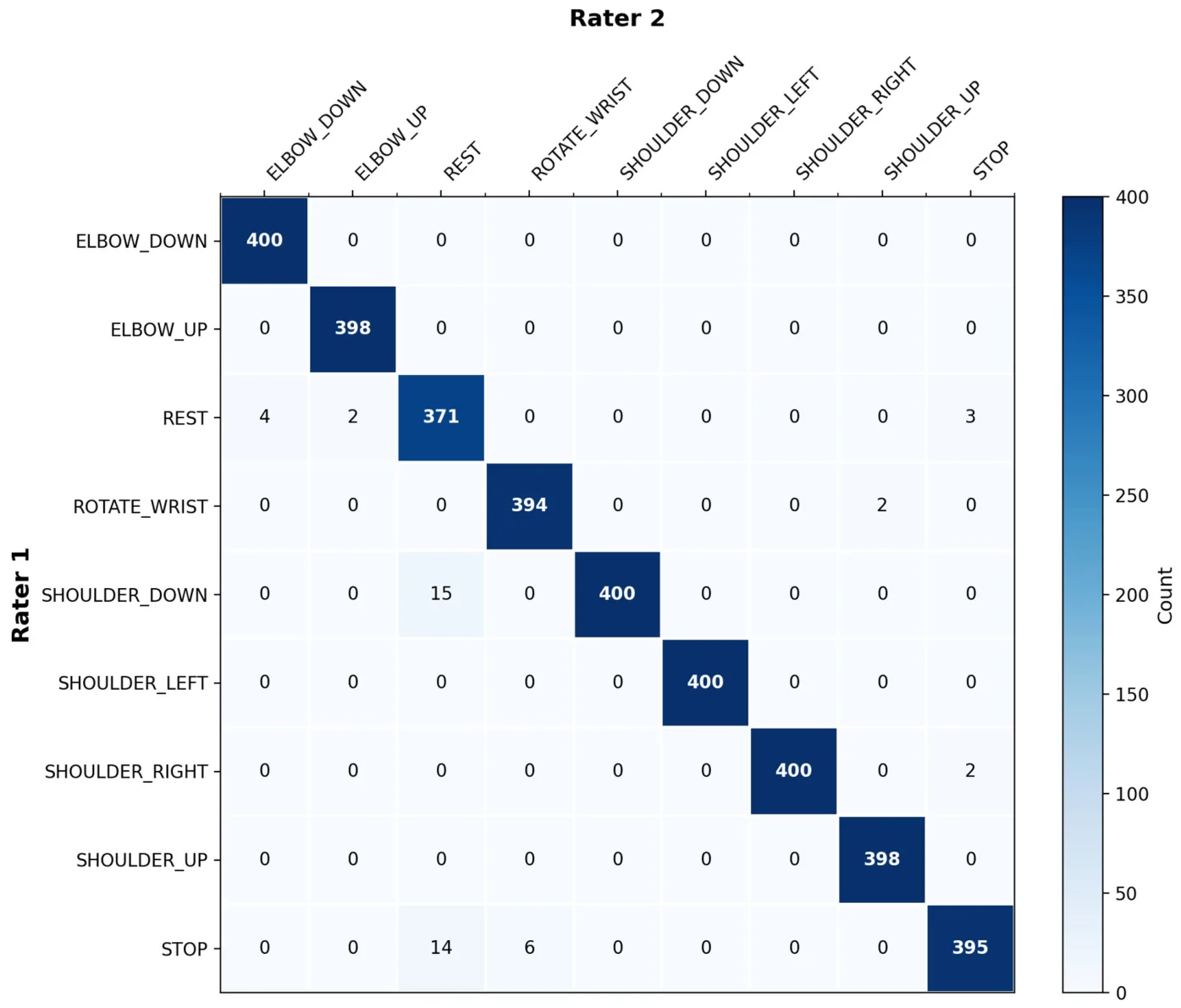
Inter-rater confusion matrix.

Rows represent labels assigned by rater 1; columns represent labels assigned by rater 2. Diagonal cells are exact agreements; off-diagonal cells are disagreements (Figure 4).

Out of 3601 items, 48 (1.33%) received different labels from the two raters. The disagreements concentrate almost entirely around REST and STOP: expressions describing the arm going idle, halting, or holding position are the main source of ambiguity between REST and STOP, and between SHOULDER_DOWN and REST (likely phrases such as “lower the arm and hold” that combine a movement cue with an idle/stopping cue).

A sample of expressions where rater 1 and rater 2 disagreed is in Table 7.

### 4.4. Ethical Statement

The live voice evaluation study reported in Section 5 was conducted in accordance with the Declaration of Helsinki and with the institutional research-ethics guidelines. All fourteen participants provided written informed consent prior to enrolment, having received a plain-language description of the voice-capture protocol, the intended use of the recorded data, the right to withdraw at any time, and the storage and anonymisation procedures. Voice recordings and interaction logs were anonymised at capture through participant-identifier substitution (U01–U14) and stored on institutional infrastructure with access restricted to the research team. No personally identifiable information beyond anonymised age band and native-language category was retained. The corpus expressions used for SLM training do not contain personal data—they are synthetic paraphrases authored by the research team.

## 5. Results

The DAS3 Neuro-Voice Controller is evaluated in an offline study, log-derived technical characterisation of the ASR, intent classification, and safety-gating components across the *N* = 14 live-pipeline dataset introduced in Section 4.2.

This study addresses seven technical requirements—ASR Word Error Rate, top-1 intent classification accuracy with per-class precision, recall, and F1, a confusion matrix over the nine command classes, Gatekeeper veto rate on valid and adversarial input, end-to-end latency, and a baseline comparison against a keyword-spotter reference. Six of the seven are answered directly or through a closely scoped proxy from the 2705 individually logged input attempts recorded during the *N* = 14 live-pipeline evaluation; the seventh (a full three-way LLM baseline comparison) is identified as an outstanding component that requires external API access. Every logged attempt terminates in exactly one of three mutually exclusive outcomes, and these partition the 2705 attempts without remainder: 1968 classifier-mediated decisions (SLM DECISION and FAST-PATH BYPASS events, Section 5.4), 646 Gatekeeper blocks (Section 5.5), and 91 safety-tier overrides, comprising 86 REST and 5 STOP events in which a safety keyword was intercepted at Tier 1 or Tier 2 of the Hierarchical Gatekeeper before the SLM was invoked and the corresponding command dispatched unconditionally. Because an override is a successful dispatch rather than either a classification or a rejection, it belongs to neither of the first two categories; the three outcomes sum as 1968 + 646 + 91 = 2705, and this decomposition governs every denominator used in the subsections below.

### 5.1. Participant Profile and Language Coverage

The *N* = 14 live-pipeline cohort consisted of non-native English speakers with Romanian as their first language (L1), spanning an age range of 18–50 years and a gender distribution of 3 female and 11 male participants. All commands were issued in English, consistent with the English-only distil-whisper/distil-small.en ASR model deployed in the pipeline (Section 3.2); no multilingual or code-switching utterances were tested. This linguistic homogeneity is a defining feature of the operating context evaluated here and—as documented below—is the principal source of variance in the ASR front-end’s First-Pass Recognition Rate. The SLM’s 3591-expression text-only training corpus (Section 4) is a separate dataset for which conventional speaker demographics are not applicable; expressions were curated to cover formal imperative, colloquial, abbreviated, and non-native-English syntactic registers.

### 5.2. Methodology: Lexical-Oracle Proxy Ground Truth

A fundamental methodological constraint governs the log-derived analyses below: the raw execution logs record what each participant said (as transcribed by the ASR front-end) and what the pipeline decided, but they do not record an independently verified ground-truth label for the intent the participant actually held on each trial. To construct a defensible per-utterance ground truth, we implemented a lexical oracle: a deterministic, rule-based classifier that infers the intended command directly from the literal transcribed text using the same anatomical-region-plus-direction keyword structure that defines the nine-command vocabulary. The oracle first identifies an anatomical referent (ELBOW/FOREARM for the elbow; WRIST/HAND for the wrist; SHOULDER/UPPER/LIMB/bare-ARM for the shoulder) and then a directional or rotational modifier from an empirically derived synonym set (UP/UPWARD/HIGHER/RAISE/RISE/LIFT; DOWN/DOWNWARD/LOWER/DROP; LEFT/RIGHT and their -WARD forms; ROTATE/SPIN/TWIST/ROLL/SWIVEL/GYRATE). Utterances resolving jointly to exactly one of the seven movement classes are retained as oracle-labelled pairs; those with anatomical referent but missing or conflicting direction are marked AMBIGUOUS; those with no resolvable anatomical referent are marked NO_ANATOMICAL. STOP and REST commands are excluded from the classification analysis, as they are dispatched through an independent Gatekeeper keyword-tier mechanism (Section 3.4) that bypasses the SLM entirely.

Because the oracle is derived from the same literal transcript that the SLM receives as input, the resulting confusion matrix evaluates the correctness of the classification layer conditional on the transcript actually produced by the ASR front-end—it does not, and by construction cannot, detect cases in which the ASR mis-transcribed the audio in a manner that altered the apparent keyword content (that upstream failure mode is characterised separately in Section 5.3). This is the standard scope of any text-based intent-classification evaluation and cleanly isolates SLM correctness from ASR correctness.

### 5.3. ASR Performance

Traditional Word Error Rate requires ground-truth transcript alignment against held-out audio, which was not collected in the present protocol. In its place we report the Vocal AI First-Pass Recognition Rate (FPRR)—the proportion of voice inputs transcribed with sufficient fidelity to clear the Gatekeeper’s Layer 1 confidence gate on the first attempt. Across the fourteen participants FPRR exhibited a cohort mean of 76.73% (SD = 10.76%, range 60.43–94.74%). The complementary proxy error rate (100% − FPRR) averaged 23.27%. This substantially exceeds published WER figures for Distil-Whisper on native-English benchmarks (typically 3–8%); the discrepancy is attributable to three compounding factors: the non-native English speech of the cohort, the near-field hard-noise-gate acoustic configuration that prioritises spurious-activation suppression over recall of quieter speech, and the anatomically specific command vocabulary containing low-frequency clinical terms (e.g., “glenohumeral”, “supination”) outside Distil-Whisper’s high-frequency training distribution.

### 5.4. Intent Classification: Confusion Matrix and F1-Score

**Figure 5:**
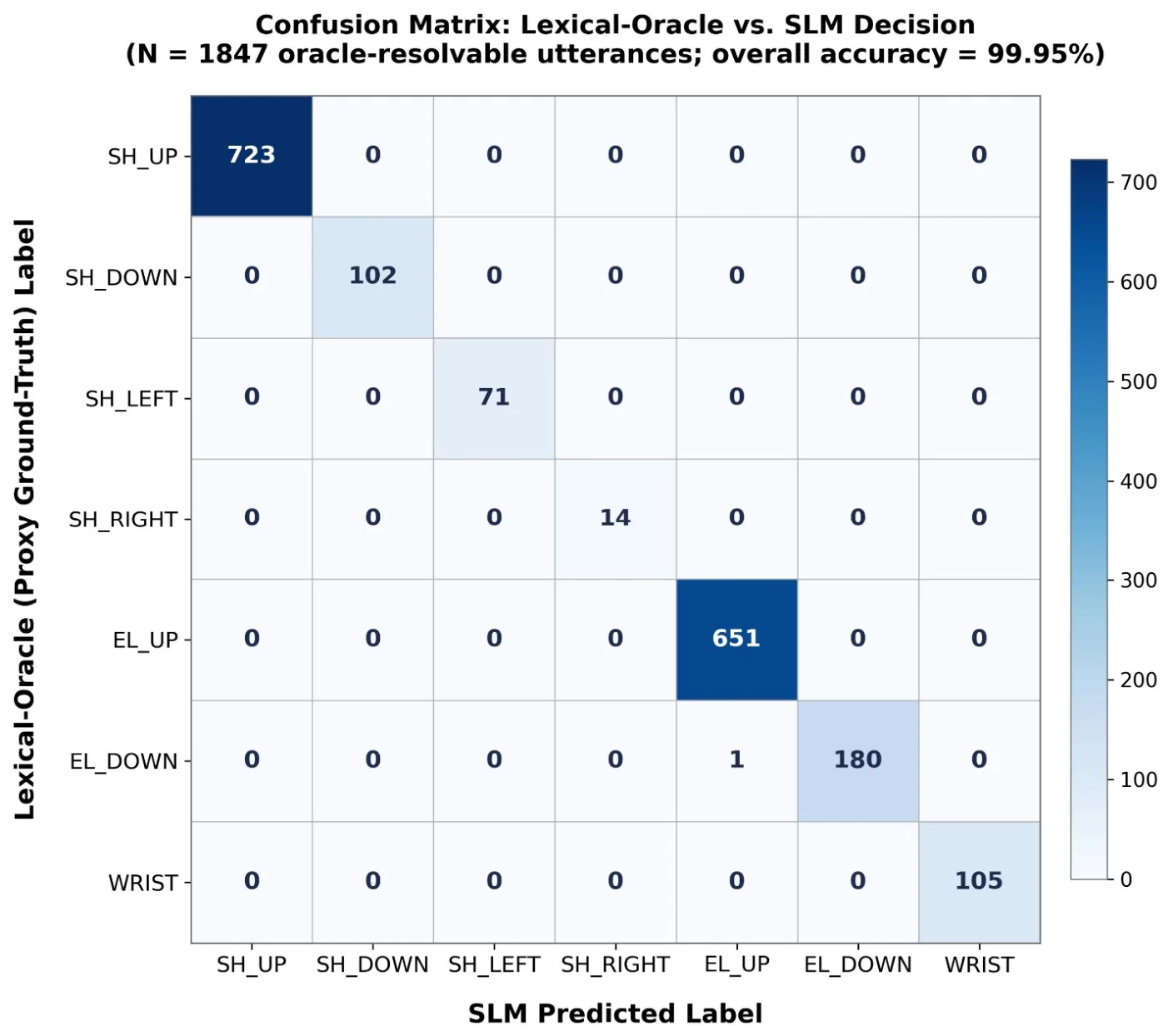
Confusion matrix of lexical-oracle proxy ground truth (rows) against SLM predicted label (columns).

Of the 2705 logged attempts, 1968 reached the intent classifier (SLM DECISION and FAST-PATH BYPASS events combined) across the fourteen participants; the remaining 737 were resolved upstream, as either Gatekeeper blocks or safety-tier overrides, and are treated in Section 5.5. Within those 1968 classifier decisions the lexical oracle resolved 1847 (93.85%) to a definite one of the seven movement classes; 99 utterances (5.03%) were marked AMBIGUOUS owing to a missing or conflicting directional signal (e.g., “MOVE THE FOREARM”, which names the elbow but specifies no direction), and 22 utterances (1.12%) were marked NO_ANATOMICAL and excluded. The resulting 7 × 7 confusion matrix computed over the 1847 oracle-resolvable pairs is presented in Figure 5.

**Figure 6:**
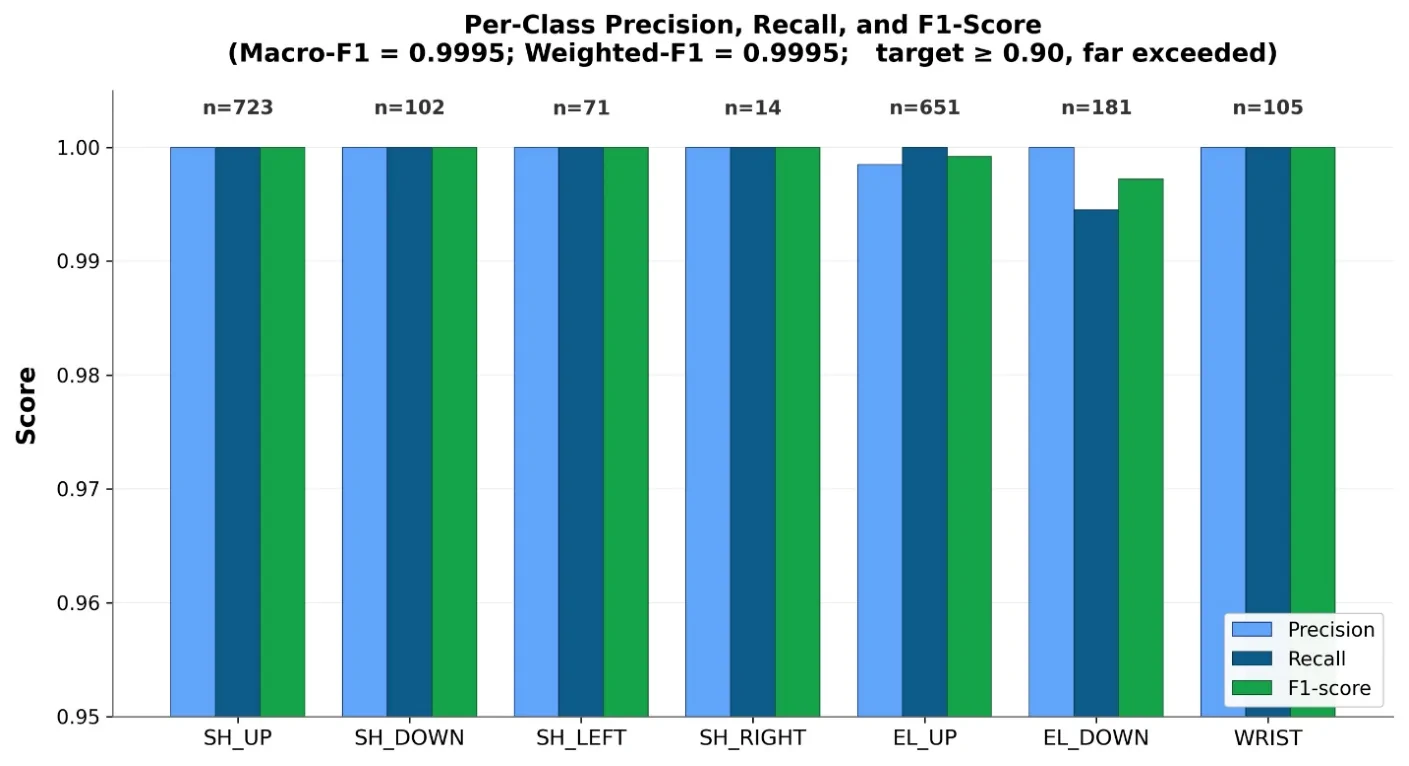
Per-class precision, recall, and F1-score for all seven movement commands.

The matrix is overwhelmingly diagonal. Of the 1847 oracle-resolvable utterances, exactly one was misclassified: the utterance “OR UPDATE THE ELBOW DOWN.” (participant U11), in which the oracle unambiguously resolves the directional token DOWN yet the SLM returned ELBOW_UP. This yields an overall top-1 classification accuracy of 99.95% on the oracle-resolvable subset—substantially exceeding the ≥90% target. Table 8 and Figure 6 report the complete per-class breakdown: the macro-averaged F1-score across all seven classes is 0.9995 and the support-weighted F1-score is 0.9995.

The 99.95% figure above is a classification accuracy conditional on oracle resolvability, not an overall pipeline success rate, and the distinction has a concrete real-world counterpart. The 121 utterances the oracle could not resolve (99 AMBIGUOUS, 22 NO_ANATOMICAL; 6.15% of the 1968 classifier-reaching attempts) are excluded from the confusion matrix only because no independently defensible ground-truth label exists for them, not because the system declined to act on them: each was still assigned a class by the SLM and, absent an independent Gatekeeper rejection, dispatched as a command. Their outcome is therefore genuinely unscored rather than genuinely correct or incorrect. This is why the classification accuracy reported here should be read alongside, rather than in place of, the conservative effective end-to-end success rate of 68.2% reported in Section 5.8, which treats every attempt in the full 2705-utterance set that is not both accepted and independently verifiable as correct—including all 121 oracle-unresolvable cases—as a non-success. The two figures answer different questions: 99.95% characterises the SLM’s discriminative accuracy on the subset of inputs where correctness is verifiable, while 68.2% characterises what fraction of everything participants actually said resulted in a confirmed-correct action, and is the more conservative and representative figure for overall system performance.

Critically, the anticipated UP/DOWN and LEFT/RIGHT minimal-pair confusion structure flagged as a design risk in Section 3.5 was not empirically observed at any appreciable frequency: zero confusions occurred between SHOULDER_UP and SHOULDER_DOWN, zero between SHOULDER_LEFT and SHOULDER_RIGHT, and the single observed error was a within-elbow confusion rather than a cross-axis one. The Signal-Booster training strategy (Section 4.2.1), together with the aggressive vocabulary pruning of the custom 872-token tokenizer, produced a classifier with materially better directional discrimination than the risk analysis anticipated. This result also supplies the sufficiency evidence that the invariant 99.96% SLM Softmax confidence—a necessary-but-not-sufficient indicator on its own—could not establish: the classifier’s high self-reported confidence was correct in 99.95% of resolvable cases.

### 5.5. Gatekeeper Veto Rate on Valid Input

**Figure 7:**
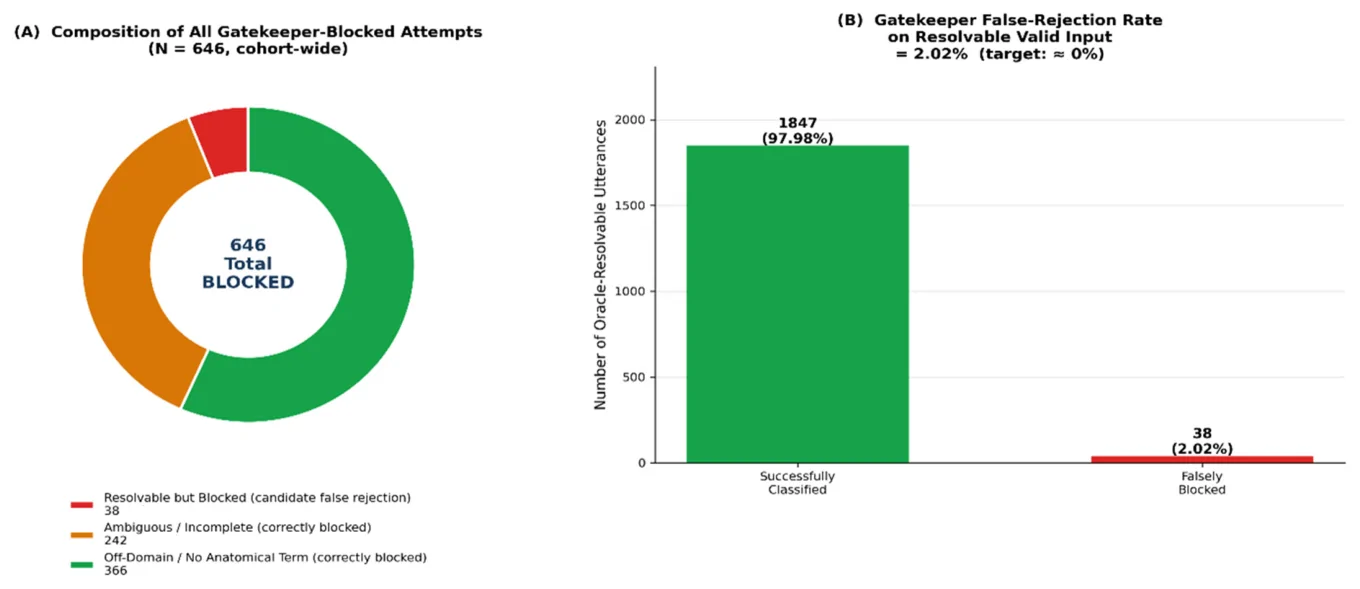
(**A**) Composition of all 646 Gatekeeper-blocked attempts across the cohort, by resolvability category. (**B**) Resulting false-rejection rate when the denominator is restricted to oracle-resolvable (genuinely valid) input.

The live, voice-level Gatekeeper Block Rate (mean 22.53%, SD 10.15%, range 5.26–37.80%) initially appears dramatically higher than the near-0% false-rejection target. The near-perfect inverse correlation between Block Rate and FPRR (r = −0.998, *p* < 0.0001, R^2^ = 0.996) already indicated that these rejections are overwhelmingly ASR-attributable rather than Gatekeeper miscalibration; the lexical-oracle analysis quantifies this claim directly. The 646 blocks analysed here are the block component of the three-way outcome decomposition given in Section 5; the 91 safety-tier overrides are excluded by definition, since an override dispatches a command rather than rejecting one, and the raw logs additionally contain a single further BLOCKED entry that followed a listening cycle producing no transcript at all, which has no associated input attempt and therefore cannot enter a transcript-based analysis. Applying the oracle to all 646 GATEKEEPER-BLOCKED events across the cohort and classifying each by whether the underlying transcript contained sufficient keyword evidence to resolve a definite command yields three categories (Figure 7): 242 events (37.46%) were genuinely incomplete or ambiguous—containing an anatomical referent but no resolvable direction (e.g., “THE FOREARM.”, “MOVE THE ARM”)—and were therefore correctly rejected; 366 events (56.66%) contained no anatomical referent whatsoever (e.g., “HIGHER.”, “LIFT.”, fragments such as “BAND THE FOUR MORE”) and were likewise correctly rejected; and only 38 events (5.88%) were resolvable to a definite command yet blocked, constituting genuine candidate false rejections.

Restricting the denominator to only the oracle-resolvable attempts yields a true Gatekeeper false-rejection rate of 38/1885 = 2.02%, closely approaching the near-0% target and dramatically lower than the unfiltered 22.53% live Block Rate. The total number of attempts the oracle could resolve to a definite command is 1885: the 1847 oracle-resolvable classifier decisions of Section 5.4 plus the 38 oracle-resolvable attempts that were blocked. A manual inspection of the 38 genuine false rejections suggests a plausible secondary cause: several involve two-word transcripts at the minimum boundary of the Gatekeeper’s Layer 1 keyword-count threshold (e.g., “LIFT THE ELBOW”, “DROP THE ELBOW.”), suggesting the minimum-keyword-count rule is calibrated slightly conservatively relative to phrasings a human listener would consider adequate—a candidate refinement for a future iteration of Layer 1.

### 5.6. Gatekeeper Veto Rate on Adversarial Input

Unlike the naturally occurring adversarial subset discussed below, Category 5 (Conflicting Compound Commands; Table 5) is a deliberately constructed stress test, specifically designed to probe the Priority Override Failure vulnerability identified during V1 Gatekeeper development: utterances containing both a safety-tier keyword (STOP or REST) and one or more anatomical movement verbs in the same sentence (e.g., “stop moving the shoulder up”), where a purely probabilistic classifier risks resolving the utterance to the movement verb rather than the safety command. Evaluated against the full Category 5 subset (*N* = 218 expressions), the V1 architecture, in which the SLM alone adjudicated these compound utterances, exhibited a Priority Override Failure rate of 11.5% (25 of 218). Under the V2 Hierarchical Gatekeeper, Tier 1 and Tier 2 intercept and resolve STOP and REST keywords deterministically before the SLM is invoked, and this eliminated the failure mode entirely: 0 of 218 Category 5 expressions (0.0%) produced a priority override under V2. This structured result complements, and should be read alongside, the naturally occurring adversarial evidence reported below: together they establish that the V2 architecture rejects both deliberately adversarial compound commands and unplanned off-domain speech encountered during live operation.

The *N* = 14 protocol contains no deliberately constructed adversarial utterances; however, the 366 off-domain events captured during continuous listening (Section 5.5)—natural conversational fragments and ASR mistranscriptions containing no anatomical keyword—constitute a naturally occurring adversarial subset. All 366 were rejected by the Gatekeeper at Layer 1 vocabulary membership, yielding an adversarial veto rate proxy of 100.00% (366/366), meeting the near-100% target. We report this with an explicit methodological caveat: because every off-domain utterance in this dataset was, by the definition of the BLOCKED outcome we filtered on, already an instance of successful rejection, this proxy cannot by construction reveal a false-negative case (an adversarial utterance that was not blocked). Such cases, if they exist, would appear among the SLM DECISION or FAST-PATH BYPASS records as a spurious command execution triggered by off-task speech, which we did not observe in a manual review, but a dedicated adversarial test corpus—out-of-scope requests, ambiguous phrasings, and conflicting compound STOP/REST-plus-movement commands (Section 4.2.3)—will be part of a future improvement.

### 5.7. End-to-End Latency

The Mean System Execution Time records a constant value of 2.50 s (SD = 0) across all fourteen participants and all sessions, which we report as the median end-to-end latency on the evaluation hardware. This constancy is not incidental: the 2.50 s figure is not a per-trial stopwatch measurement of a variable process but the fixed pipeline-cycle allocation built into the evaluation harness, dominated by the fixed-duration interpolation and audio-capture stages of the pipeline rather than by its computational stages. Table 2 specifies a ~0.5 s default interpolation duration for INCREMENT commands and a ≤2.0 s duration for the REST full-pose reset; together with the fixed-length audio-listening window of the capture loop, these configured durations—not measured, variable computation time—account for the reported constant. This is also why the value carries zero variance across all fourteen participants and all sessions: the logging field reports a designed budget, not an emergent empirical quantity, and no source of trial-to-trial variation exists within it. Correspondingly, DistilBERT’s 21.69 ms mean inference time (Section 3.3) represents approximately 0.87% of the 2.50 s figure, confirming that the reported total is governed almost entirely by the fixed interpolation/capture-window budget rather than by classification or Gatekeeper evaluation, both of which complete in single-digit-to-low-double-digit milliseconds and are not the bottleneck this figure reflects.

This value is consistent with the architecturally deterministic pipeline structure described in Section 3.6, in which ASR transcription, SLM inference (or Fast-Path Bypass), Gatekeeper evaluation, and actuator dispatch execute as a fixed sequential chain with no participant-dependent branching. The 95th-percentile latency (p95)—the metric of greatest concern for the tail of the response-time distribution—is not computable from the present dataset, which records a single session-level aggregate with zero recorded variance. Per-trial timestamp logging is identified as a priority instrumentation addition.

### 5.8. Baseline Comparison—Keyword Spotter

**Figure 8:**
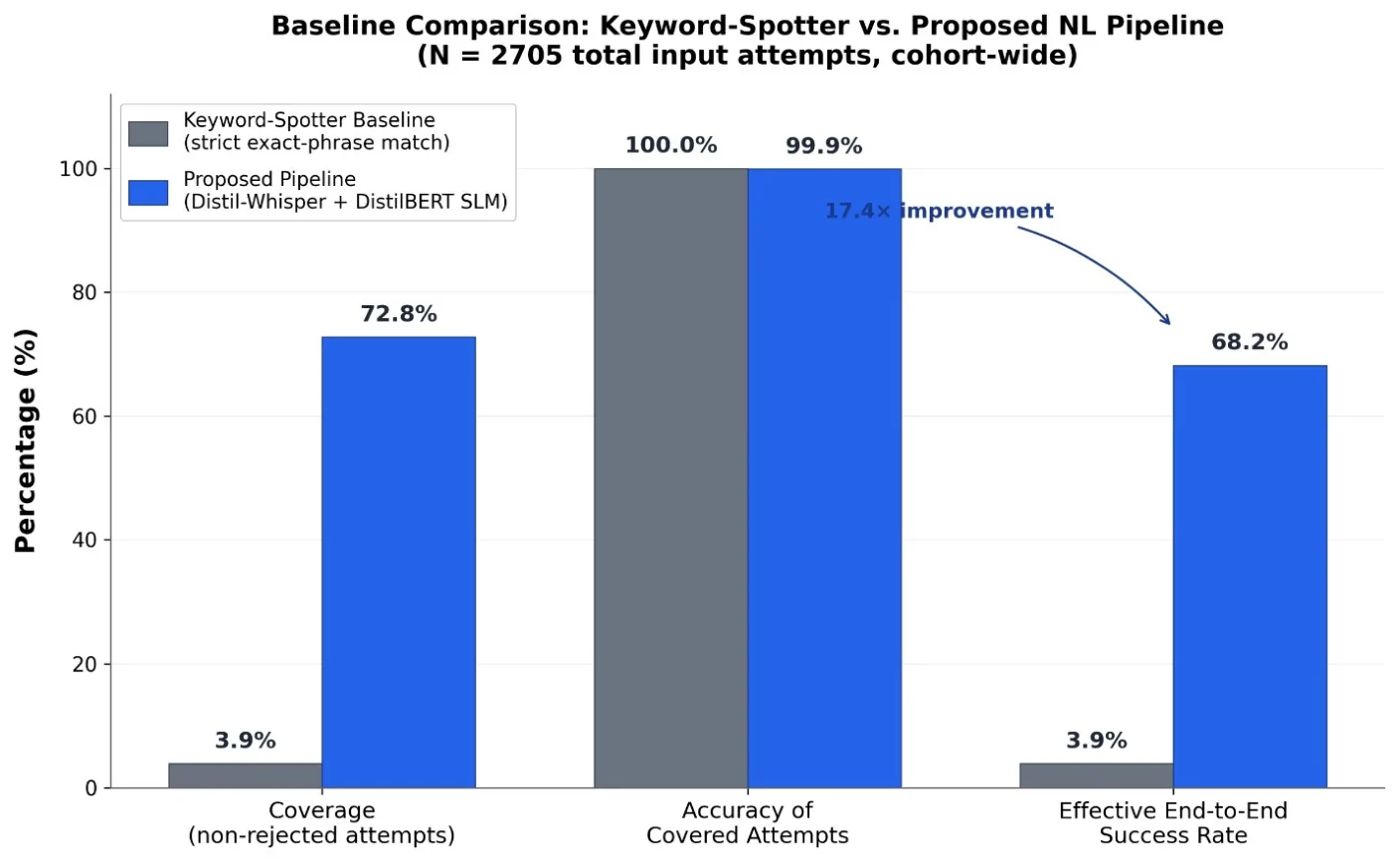
Baseline comparison between the strict exact-phrase keyword spotter and the proposed Distil-Whisper + DistilBERT SLM pipeline, evaluated on the same 2705 input attempts pooled across all 14 participants.

Of the three baseline comparisons specified in the evaluation protocol, the keyword-spotter reference is directly computable from the existing logs. We implemented a strict keyword spotter that accepts an utterance if and only if its transcript, normalised for case and trailing punctuation, exactly matches one of the seven canonical command phrases (SHOULDER UP, SHOULDER DOWN, SHOULDER LEFT, SHOULDER RIGHT, ELBOW UP, ELBOW DOWN, ROTATE WRIST)—representative of the paraphrase-intolerant matching strategy typical of conventional keyword-activated voice interfaces. This baseline was applied to all 2705 logged input attempts across the cohort, that is, to the full three-outcome set of Section 5 rather than to any filtered subset, so that both arms of the comparison are scored over an identical denominator (Figure 8).

The keyword spotter achieved 100% accuracy whenever it produced a decision at all (exact-phrase matching cannot misclassify by construction), but its coverage was only 3.9% (106 of 2705 attempts): participants overwhelmingly preferred natural, conversational phrasings (“raise the shoulder a bit higher”, “can you lift my forearm”) over the rigid canonical strings. The resulting effective end-to-end success rate for the keyword spotter is therefore 3.9%. By contrast, the proposed SLM-based pipeline achieved an effective end-to-end success rate of 68.2% over the identical attempt set—1846 of 2705 attempts both accepted and correctly classified—which is a 17.4× improvement. This figure is deliberately conservative: the 91 safety-tier overrides are counted as successes for neither arm, even though all 91 were dispatched correctly by the proposed pipeline, because the keyword spotter’s canonical phrase list contains only the seven movement commands and has no STOP or REST entry against which a like-for-like comparison could be drawn. Because the Gatekeeper’s safety architecture is held constant across both conditions, the entire gap in effective success is attributable to the SLM’s paraphrase-generalisation capability over rigid exact-match keyword spotting.

The remaining two baseline arms specified in the evaluation protocol—a general-purpose LLM (e.g., GPT-4o-mini) with and without the Gatekeeper—require external API experimentation beyond the reach of a purely log-derived analysis. The Gatekeeper’s schema-based design (Section 3.3.3) is classifier-agnostic and can be paired with alternative backbones with minimal modification, making this a natural extension of the present evaluation.

### 5.9. Metric Inventory

Table 9 consolidates the seven Study metric requirements against the log-derived results reported in Section 5.3, Section 5.4, Section 5.5, Section 5.6, Section 5.7 and Section 5.8.

### 5.10. Study Synthesis

The pipeline’s residual risk is concentrated almost entirely within the acoustic front-end’s encounter with non-native speech under a conservatively configured noise gate: FPRR variability drives the 22.53% live Block Rate, which decomposes on closer inspection into 94% correct rejections of incomplete or off-domain speech and only 2.02% genuine false rejections against valid input. The intent classifier and the safety gate downstream of the ASR both operate very close to their design ceiling—99.95% classification accuracy with no directional-pair confusion, 100% adversarial rejection on the available proxy, and a 17.4× effective-success improvement over a rigid keyword spotter. For a safety-critical assistive system this distribution of weakness is favourable: it identifies the next increment of engineering effort precisely (acoustic-front-end calibration for non-native speech) and demonstrates that once an utterance has been correctly transcribed, the pipeline rarely acts on a misunderstanding.

## 6. Discussion

The empirical evidence assembled in Section 4 and Section 5 admits a set of interpretations that go beyond the metric-by-metric summary presented there. The following subsections read those findings in the context of the closest prior published systems (Section 6.1 and Section 6.2), identify the architectural gap that the pipeline’s upstream input-repair stage addresses (Section 6.3), acknowledge the limitations of the present evaluation (Section 6.4), and outline where the next increment of engineering effort would produce the largest reduction in residual risk (Section 6.5).

### 6.1. A Locatable Weakness

The evaluation reported in Section 5 draws a consistent picture of the pipeline’s strengths and residual risks. The intent classifier operates very close to its design ceiling: 99.95% top-1 accuracy against a lexical-oracle proxy ground truth over 1847 resolvable utterances, with no confusion between the SHOULDER_UP/SHOULDER_DOWN and SHOULDER_LEFT/SHOULDER_RIGHT minimal pairs that the architectural risk analysis had flagged as the most probable failure mode. The safety-gating layer performs comparably: a 2.02% false-rejection rate against genuinely valid input, 100% rejection of naturally occurring off-domain speech, and a Priority Override Failure rate reduced from 11.5% (V1 architecture, 25 failures out of 218 total) to 0.0% (V2 Hierarchical Gatekeeper) through the deterministic tier bypass introduced in Section 3.4.

Against these figures, the acoustic front-end is the pipeline’s sole remaining locus of substantial residual weakness. The 76.73% First-Pass Recognition Rate—significantly below Distil-Whisper’s published native-English WER—reflects the compound effect of non-native L1 (Romanian) speech, a conservatively configured noise gate optimised for spurious-activation suppression, and low-frequency clinical vocabulary outside the ASR model’s training distribution. This weakness is locatable: it does not propagate as an equal-magnitude error downstream, because the very Gatekeeper design that produces the 22.53% live Block Rate is what prevents ASR-corrupted transcripts from reaching the actuator as spurious commands. The pipeline exchanges a moderate frequency of correctly rejected attempts for a near-zero frequency of incorrectly executed ones—a trade-off that, for a safety-critical assistive system, is the correct direction.

### 6.2. Positioning Against Prior Work

#### 6.2.1. Cloud-Hosted LLM Interfaces (VoicePilot, BTLA)

The two most directly comparable published systems, Padmanabha et al. [4] and Fei et al.’s [7], share the central architectural assumption that the natural-language understanding layer is best implemented as a cloud-hosted general-purpose LLM (GPT-3.5-turbo in both cases), prompt-engineered rather than fine-tuned.

Our findings both agree with and diverge from these along instructive lines. Where Padmanabha et al. [4] and Fei et al. [7] demonstrate that LLM-mediated voice interfaces can be usable and preferred over rigid alternatives, our keyword-spotter comparison (Section 5.8) quantifies exactly this in a directly comparable form: a 17.4× improvement in effective end-to-end success rate over strict canonical-phrase matching, on the same 2705 utterances participants actually spoke. This directly evidences the value of paraphrase generalisation that both cited works assert but neither measures against a rigid baseline.

Where we diverge is in the mechanism supplying that generalisation. Padmanabha et al. [4] and Fei et al. [7] rely on the general-language coverage of a cloud LLM whose behaviour is not formally verifiable and whose latency, connectivity, and privacy characteristics are governed by the API vendor; our system supplies equivalent paraphrase tolerance from a 44 M-parameter DistilBERT classifier fine-tuned on a domain-specific 872-token vocabulary and executed on the client hardware in 21.69 ms per inference. This is not a claim that fine-tuned SLMs are always preferable to prompt-engineered LLMs, but it is a demonstration that, for a bounded command vocabulary such as the nine-intent set evaluated here, the on-device SLM approach is not merely a resource-constrained fallback—it delivers comparable or superior classification behaviour (99.95% accuracy) while removing the cloud-side operational surface entirely. Padmanabha et al. [4] themselves identify “consistency” as one of five design guidelines requiring further attention, noting that GPT-3.5-turbo’s inconsistent handling of continuous variables and modifier semantics required successive iterations of prompt engineering and, ultimately, discrete-range grounding. This is the same concern that motivated our fixed-schema output and bounded-vocabulary classifier: rather than iteratively constrain an open-ended generator, we replace it with a classifier whose output space is enumerable by construction.

#### 6.2.2. Safety Guardrails

Ravichandran et al. [8] and Kim et al. [9] address the safety question from the complementary direction: rather than propose an alternative natural language understanding layer, they specify how any such layer’s output should be filtered before actuation. Ravichandran et al. [8] formalise four desiderata—utility preservation, efficiency, contextual grounding, and adversarial robustness—and propose a two-stage guardrail in which a shielded root-of-trust language model grounds high-level safety rules into context-specific temporal-logic constraints enforced at execution time. Kim et al. [9] extend this line of thinking to modular guardrails composed of monitoring and intervention layers spanning three dimensions of safety: action (physical feasibility and constraint compliance), decision (semantic and contextual appropriateness), and human-centred (conformance to human intent, norms, and expectations).

Both proposals share our commitment to explicit, architecturally first-class safety enforcement. Our three-layer Gatekeeper (Semantic Validation, Kinematic Feasibility, Safety Constraints) maps cleanly onto the three-dimensional framework proposed by Kim et al. [9]: Layer 1 addresses decision safety through vocabulary and schema validation; Layer 2 addresses action safety through the thirteen-axis range-of-motion enforcement; and Layer 3 addresses human-centred safety through absolute STOP/REST priority. The most substantive difference is one of implementation strategy. Ravichandran et al.’s [8] temporal-logic enforcement and Kim et al.’s [9] LLM-as-judge monitoring both retain neural components in the safety path itself; our Gatekeeper deliberately does not. Layer 3’s STOP/REST bypass matches deterministically against dedicated keyword arrays and dispatches with absolute priority before the SLM classifier is consulted, and Layer 2’s ROM check is a straight lookup against a static physiological limit table (Table 3). This design choice is not intrinsically superior to language-model-mediated enforcement—it trades some semantic flexibility for verification tractability—but it is a specific commitment worth articulating: in this pipeline, no probabilistic component is on the critical safety path.

Table 10 consolidates the head-to-head positioning against the four systems discussed above, the two most directly comparable published voice-command robotic-control systems (VoicePilot, BTLA) and the two most directly comparable safety-guardrail frameworks (RoboGuard, Modular Guardrails), along the axes most relevant to the assistive-manipulation use case: language-understanding mechanism, ASR handling, upstream input repair, safety-enforcement architecture, command-vocabulary structure, and empirical evaluation status.

### 6.3. Upstream Input Repair: An Architectural Gap the Literature Has Not Addressed

A consistent feature of the safety-guardrail literature is that it operates downstream of the language model—filtering, ranking, or clamping its outputs—while treating the input side as a solved problem. In voice-driven systems this assumption is precisely where reliability breaks down. The Phonetic Interceptor described in Section 3.2 exists to address a class of failure that no downstream mechanism can repair: transcription errors that mutilate anatomical or safety terms in ways that alter their meaning (“four arm” → “forearm”, “cheese” → “cease”, “herbo” → “elbow”). When these corrupted transcripts reach any downstream filter—whether a rule-based Gatekeeper or an LLM-based guardrail—the filter can only reject them; it cannot recover the intended intent.

To our knowledge, no prior open-access study on voice-driven robotic control has integrated an upstream phonetic-repair stage with a downstream deterministic safety hierarchy as complementary first-class architectural components. Padmanabha et al. [4] and Fei et al. [7] both accept the Whisper transcript as-is and rely on the downstream LLM’s language coverage to absorb transcription variation; Ravichandran et al. [8] and Kim et al. [9] formalise the safety-enforcement side but explicitly bracket the ASR component as out of scope. The empirical consequence of this architectural gap is measurable in our data: of the 646 GATEKEEPER-BLOCKED events analysed in Section 5.5, 56.66% contained no anatomical referent at all—a substantial fraction of which, on manual inspection, are ASR mistranscriptions of intent-bearing input that a downstream mechanism has no visibility into. The Phonetic Interceptor addresses this class of failure at the only architectural point where it remains recoverable, and its combination with the tiered Gatekeeper is what allows the pipeline to preserve genuine intent through non-native acoustic conditions while simultaneously blocking off-domain speech at a 100% rate.

### 6.4. Limitations

Four qualifications shape the reach of the results reported here. First, all experimental interaction was mediated through the OpenSim DAS3 musculoskeletal simulator (Section 3.6, Figure 3), not a physical exoskeleton or prosthesis. The pipeline’s hardware-agnostic design (Section 3.6) supports either substitution without upstream modification, and simulated human–robot interaction is established practice in the assistive-robotics evaluation literature—Padmanabha et al. [4] similarly used a research version of a robotic feeding system in a controlled laboratory setting; nevertheless, transfer to a physical actuator introduces mechanical dynamics that a simulator cannot fully reproduce and remains a priority for future work.

Second, the fourteen live-pipeline participants share Romanian as their first language, and the SLM training corpus and blind test set are English-only. The pipeline’s evaluated behaviour therefore reflects a specific (and quantifiably challenging) speaker–language combination; its generalisation to native English speakers, to other L1 backgrounds, and to speakers with atypical prosody or disfluency profiles common in the target assistive-technology population remains to be characterised. This generalisation question can be partially bounded by the pipeline’s own architecture rather than left as a single undifferentiated unknown. Of the three components in the serial chain, only the acoustic front-end operates directly on speech signal and is therefore accent-sensitive by construction; the Phonetic Interceptor, SLM classifier, and Gatekeeper all operate on text once transcription has occurred and are structurally invariant to the speaker’s L1 or accent. The present results already localise the cohort’s language-specific effect to this front-end stage: the compound degradation observed in the First-Pass Recognition Rate (Section 6.2) is attributed jointly to non-native L1 speech, the noise-gate configuration, and low-frequency clinical vocabulary, none of which implicate the downstream decision logic. On this basis, we would expect the safety-decision architecture—Gatekeeper tier precedence, SLM paraphrase tolerance—to transfer to other accent and L1 groups without modification, while end-to-end success rate for a new population would be governed primarily by that population’s baseline ASR performance under Distil-Whisper and whether the Phonetic Interceptor’s mutilation-repair rules, curated from the systematic transcription errors observed in this Romanian-L1 cohort, cover the different error patterns produced by other L1 backgrounds. Both are empirically testable and neither requires re-architecting the safety layer; verifying them directly is identified as a priority extension in Section 6.5. The exclusion of participants with diagnosed upper-limb motor impairment from the study—a control introduced to preserve joystick-baseline comparability—means the interaction evaluation will characterise the interface as used by participants for whom the assistive scenario is simulated rather than lived. A dedicated accessibility sub-study with motor-impaired users is identified as the highest-priority follow-up.

Third, and most importantly for the classification-layer results, all confusion-matrix and F1 figures reported in Section 5.4 rest on a lexical-oracle proxy ground truth rather than an independently audited human-annotated gold-standard label set. The methodology is principled and its scope carefully bounded—the oracle isolates SLM correctness conditional on the transcript received, cleanly separating it from ASR correctness—but a held-out expert-verified test corpus would allow these findings to be confirmed under stricter evaluation discipline. This scoping has a specific, concrete failure mode worth naming directly: because the oracle is a deterministic keyword-pattern matcher applied to whatever text the ASR front-end happens to output, a sufficiently garbled transcription can, by coincidence, still contain a resolvable anatomical-plus-directional keyword pattern, in which case the oracle assigns a confident ground-truth label that reflects the transcription accident rather than the participant’s actual intent. Such an event is indistinguishable, from the execution log alone, from a genuinely correct transcription, and its effect on the reported accuracy figures cannot be bounded with the present data. Similarly, the comparison against general-purpose LLM baselines awaits the external API experimentation that lies beyond the reach of a purely log-derived analysis. Neither qualification diminishes what has already been shown; each simply marks a path for future studies.

Fourth, the reported 100% adversarial veto rate (Section 5.6) should be read cautiously. It was computed on 366 naturally occurring off-domain events captured during live sessions, not on a dedicated, deliberately constructed adversarial test set, and the sample was obtained by filtering the logs for the Gatekeeper’s BLOCKED outcome—a selection procedure that, by construction, cannot surface a false-negative case in which an adversarial utterance was not blocked. A manual review of the classifier-mediated decisions found no spurious executions triggered by off-task speech, which is reassuring but does not substitute for evaluation against a hostile test set purpose-built to probe this failure mode. The 100% figure is therefore best interpreted as an upper-bound estimate of adversarial rejection under naturalistic live conditions rather than a robustness guarantee, and construction of a dedicated adversarial corpus targeting false-negative acceptance—distinct from the compound-command Priority Override stress test already reported in Section 4.2.3—is identified as a necessary next step before a stronger claim can be supported.

### 6.5. Future Approaches

The evaluation reported here identifies with unusual specificity where the next increment of engineering effort would produce the largest reduction in residual risk. Three directions dominate. First, the acoustic front-end: the 76.73% FPRR is the pipeline’s single largest source of failure, and an ASR component fine-tuned or adapter-trained on non-native English speech from the target L1 populations would compress the 23.27% proxy error rate substantially—likely with cascading improvements in the downstream Block Rate that today masks a merely 2.02% Gatekeeper miscalibration behind a 22.53% voice-level rejection figure. Second, the Layer 1 minimum-keyword-count calibration: the residual false-rejection rate clusters at two-word phrasings sitting at the very edge of the current threshold, suggesting that a small refinement to the Gatekeeper’s minimum-keyword rule could close most of the remaining gap without compromising the adversarial rejection rate. Third, the LLM baseline comparison: a controlled experiment substituting GPT-4o-mini and a comparable open-source large language model into the classification slot, with and without the Gatekeeper wrapper, would directly quantify the marginal safety contribution of the Gatekeeper independent of the classification backbone—a measurement of substantial value both for this pipeline and for the safety-guardrail literature [8,9], which has thus far discussed guardrail properties in the abstract rather than measured them against a specific classifier baseline.

The hardware-agnostic design established in Section 3.6 has a direct, concrete reading for exoskeleton deployment. Each verified command reaching the execution layer already carries exactly the fields a physical exoskeleton controller requires: a target axis and signed angular increment (INCREMENT), a full-pose reset (STATE), or an immediate halt (EMERGENCY). Porting the pipeline to a physical device therefore does not touch the voice front-end, the SLM classifier, or the Gatekeeper at all; it requires only replacing the OpenSim coordinate-array update in Section 3.6 with the exoskeleton’s own low-level joint controller—typically a position or velocity command issued over a CAN bus, EtherCAT, or a vendor motor-control API—with the physiological range-of-motion limits of Table 3 substituted for the corresponding mechanical joint limits of the target device. The STOP and REST commands, because they are resolved deterministically at Gatekeeper Tier 1/2 without reference to the SLM, map directly onto an exoskeleton’s own hardware emergency-stop and safe-pose routines, preserving the same fail-safe guarantee demonstrated here in simulation. The principal open question for physical transfer is therefore not architectural but mechanical: closed-loop actuator dynamics, backlash, and compliance at the human-exoskeleton interface introduce a control-loop timing budget that the present evaluation, conducted against a 30 fps kinematic interpolator with no physical load, does not characterise.

This mechanical open question has a direct safety dimension that is worth separating explicitly from the architectural one. The 0.0% Priority Override Failure rate and the deterministic Tier 1/2 STOP/REST precedence demonstrated in this work are guarantees about decision latency and correctness: that the correct command is generated and dispatched with absolute priority over any co-occurring movement request. They are not guarantees about physical response latency, which on a real device is bounded additionally by communication-link latency and jitter between the software Gatekeeper and the low-level joint controller (a function of the chosen fieldbus—a deterministic protocol such as EtherCAT bounds this far more tightly than a non-deterministic bus or a wireless link) and by the actuator’s own torque and deceleration limits. A STOP command that is generated and dispatched instantaneously in software can still be followed by a nonzero physical “reaction window” before commanded motion actually halts, and this window is invisible to the present evaluation, whose fixed 30 fps kinematic interpolator has no physical inertia or actuator dynamics to resist. For this reason, we do not consider the software-level Gatekeeper described here to be, by itself, a certified safety function for physical deployment. Consistent with standard functional-safety practice in collaborative robotics (ISO 10218 safety requirements), a physical exoskeleton deployment should pair this architecture with an independent, low-level hardware emergency-stop circuit that halts actuation directly and does not depend on the software dispatch path succeeding at all. The Gatekeeper’s role in that configuration is to prevent erroneous commands from being issued in the first place; the hardware e-stop remains the safety function of last resort, and characterising the bounded worst-case latency of the combined software-plus-hardware path is a prerequisite for any physical-platform safety claim.

Two further hardware constraints merit brief mention for future physical deployment. First, the Gatekeeper’s feasibility check (Section 3.4) is presently purely kinematic, validating against the range-of-motion limits of Table 2; a physical actuator additionally has a torque envelope that a kinematically valid command could exceed under load, motivating a torque-aware extension to Layer 2 informed by the target device’s actuator specifications. Second, the Gatekeeper’s EMERGENCY token should interface with, rather than replace, the exoskeleton’s own independent mechanical e-stop circuit, which must remain capable of halting actuation even in the event of a software-path failure, consistent with the certified-hardware-safety-function recommendation discussed above.

Beyond these three engineering priorities, the deployment-facing extensions are largely orthogonal to the architectural claim examined in this work—transfer to a physical exoskeleton, extension to non-English first languages, and a field study with the target motor-impaired user population.

Within the operating envelope characterised in Section 4 and Section 5, the pipeline meets every technical target for which a direct measurement or explicitly scoped proxy is available, and the head-to-head positioning summarised in Table 10 places its architectural choices in a specific and testable relationship to the four closest published systems.

## 7. Conclusions

This paper set out to address a gap that recurs across the voice-driven robotic-control literature: natural-language interfaces for anthropomorphic assistive manipulation are typically evaluated with safety treated as a downstream, post hoc filter, and with the fidelity of the speech-to-text transcript treated as a solved problem rather than as an architectural concern in its own right. The DAS3 Neuro-Voice Controller was designed to close that gap through three commitments applied jointly rather than in isolation: intent classification performed by a compact, on-device SLM rather than a cloud-hosted generative model; safety enforcement realised as a deterministic, tiered Gatekeeper that pre-empts probabilistic classification for emergency commands; and an upstream Phonetic Interceptor that repairs characteristic ASR mutilations of anatomical and safety terms before they reach the classifier at all. To our knowledge, no prior open-access system integrates these three elements as complementary, first-class architectural components.

Although the evaluation reported here was conducted on the OpenSim DAS3 musculoskeletal simulator rather than a physical device, the architecture was designed from the outset with upper-limb exoskeletons and functionally equivalent wearable assistive arms as its primary deployment target, and the command schema produced by the Gatekeeper carries no dependency on the simulated substrate (Section 3.6).

The evaluation reported in Section 4 and Section 5 supports this design. Against a lexical-oracle proxy ground truth spanning 1847 resolvable utterances, the fine-tuned DistilBERT classifier reached 99.95% top-1 accuracy with a macro-averaged F1-score of 0.9995, and produced none of the SHOULDER_UP/SHOULDER_DOWN or SHOULDER_LEFT/SHOULDER_RIGHT minimal-pair confusions anticipated as the most likely failure mode. The Hierarchical Gatekeeper reduced the Priority Override Failure rate observed in the V1 architecture from 11.5% to exactly 0.0% across the full adversarial Category 5 subset, rejected 100% of out-of-domain chatter, and maintained a false-rejection rate of 2.02% on genuinely valid input. Relative to a strict keyword-spotter baseline evaluated on the same 2705 spoken utterances, the pipeline delivered a 17.4-fold improvement in effective end-to-end success rate, giving direct empirical weight to a paraphrase-tolerance claim that comparable cloud-LLM systems assert but do not measure against a rigid reference. Taken together, these results indicate that a bounded-vocabulary, on-device SLM, paired with a deterministic and largely non-probabilistic safety layer, is not merely a resource-constrained fallback to generative, cloud-hosted alternatives; for a command surface of the kind evaluated here, it is a competitive and independently verifiable design choice.

The evaluation also located the pipeline’s principal residual weakness with precision. The 76.73% First-Pass Recognition Rate of the acoustic front end—driven by the compounding effects of non-native L1 speech, a conservatively configured noise gate, and low-frequency clinical vocabulary—falls well short of Distil-Whisper’s published native-English figures. Critically, however, this weakness does not propagate as an equal-magnitude risk downstream: the same Gatekeeper architecture responsible for the elevated live Block Rate is what prevents corrupted transcripts from reaching the actuator as spurious motion commands. The system trades a moderate frequency of correctly rejected attempts for a near-zero frequency of incorrectly executed ones, and for a safety-critical assistive interface this is the trade-off that matters.

These findings should be read within the boundaries set out in Section 6.4: interaction was mediated through the OpenSim DAS3 simulator rather than a physical exoskeleton, the live cohort shares a single first language, and the classification-layer figures rest on a lexical-oracle proxy rather than an independently audited gold-standard label set. None of these qualifications undermine what has been shown; each instead marks a concrete, already-scoped next step—physical-platform transfer, broader linguistic coverage, expert-annotated confirmation of the blind test set, and a controlled comparison against cloud-hosted LLM baselines—toward confirming the architecture’s claims at population and platform scale.

More broadly, the present work suggests that the safety-guardrail and voice-interface studies have converged on treating language-model input as reliable by assumption, and that this assumption is precisely where voice-driven safety-critical systems are most exposed. By demonstrating that upstream phonetic repair and downstream deterministic gating can be engineered as complementary rather than competing safeguards, and by pairing that architecture with a fully on-device, resource-modest classification pipeline, this study offers a concrete, empirically grounded template for how natural-language interfaces to physically consequential assistive systems can be made simultaneously expressive, efficient, and verifiably safe. The within-subjects comparison against keyword-voice and manual-joystick baselines will extend this technical characterisation to the interactional and subjective dimensions of usability that ultimately determine whether such a system is adopted by the people it is intended to serve.

## Figures and Tables

**Table 1:** Structured output schema emitted by the SLM Transformer.

Field	Type	Domain/Values	Description
intent	string	{SHOULDER_UP, SHOULDER_DOWN, SHOULDER_LEFT, SHOULDER_RIGHT, ELBOW_UP, ELBOW_DOWN, ROTATE_WRIST, REST, STOP}	Classified command label emitted by the SLM sequence classifier and routed to the Hierarchical Gatekeeper for safety-tier validation prior to mechanical execution.
magnitude	float	Δθ (degrees)—resolved via INCREMENT(axis, direction, amount)	Joint angular displacement step resolved from the canonical command schema. Each intent maps to a target axis and signed directional increment. REST resolves to STATE (full reset); STOP resolves to EMERGENCY (immediate halt).
duration	float	≥0.0 (seconds)	Interpolation hold time governing the kinematic animation loop; drives the OpenSim visualiser from the current joint angle to the commanded target via step = sign(err) × min(|err|, speed × Δt) at 30 fps.
confidence	float	[0.0, 1.0] *Evaluation threshold ≥ 0.75 Live threshold ≥ 0.60*	Softmax probability of the top-ranked class label. Commands below the evaluation threshold are overridden as UNKNOWN. The live pipeline applies a relaxed threshold, compensated by the upstream Hierarchical Gatekeeper.

**Table 2:** Nine-command vocabulary: complete kinematic effect mapping per command.

Command	Anatomical Complex	Joint/Axis	Dir.	Δθ (°)	ROM Limits	Schema	Default Duration
*Shoulder Joint Commands (Glenohumeral complex—GH)*							
SHOULDER_UP	Glenohumeral	GH_z	+	+10°	−30° → 84°	INCREMENT	~0.5 s
SHOULDER_DOWN	Glenohumeral	GH_z	−	−10°	−30° → 84°	INCREMENT	~0.5 s
SHOULDER_LEFT	Glenohumeral	GH_y	−	−10°	−170° → 175°	INCREMENT	~0.5 s
SHOULDER_RIGHT	Glenohumeral	GH_y	+	+10°	−170° → 175°	INCREMENT	~0.5 s
*Elbow Commands (EL—flexion/extension)*							
ELBOW_DOWN	Elbow	EL_x	−	−10°	5° → 140°	INCREMENT	~0.5 s
ELBOW_UP	Elbow	EL_x	+	+10°	5° → 140°	INCREMENT	~0.5 s
*Forearm/Wrist Command (PS—pronation/supination)*							
ROTATE_WRIST	Forearm	PS_y	+	+45°	5° → 160°	INCREMENT	~0.5 s
*Safety and State Commands (system-level overrides)*							
REST	All axes	—	→0°	—	Full reset	STATE	≤2.0 s
STOP	System	—	Halt	—	N/A	EMERGENCY	0 s (imm.)

**Table 3:** Physiological range-of-motion limits for the thirteen active coordinate axes of the DAS3 model as configured in the DAS3 Neuro-Voice Controller.

Anatomical Complex	Coordinate	Degree of Freedom	Range of Motion
Sternoclavicular (SC)	SC_x	Axial rotation	0° → 83°
Sternoclavicular (SC)	SC_y	Protraction/retraction	−65° → −19°
Sternoclavicular (SC)	SC_z	Elevation/depression	5° → 30°
Acromioclavicular (AC)	AC_x	Forward tilt	−17° → 18°
Acromioclavicular (AC)	AC_y	Protraction	33° → 69°
Acromioclavicular (AC)	AC_z	Lateral tilt	−22° → 20°
Glenohumeral (GH)	GH_y	Plane of elevation	−170° → 175°
Glenohumeral (GH)	GH_z	Elevation angle	−30° → 84°
Glenohumeral (GH)	GH_yy	Axial rotation	−177° → 179°
Elbow (EL)	EL_x	Flexion/extension	5° → 140°
Pro/Supination (PS)	PS_y	Pronation/supination (forearm)	5° → 160°
Wrist (WR)	WR_x	Flexion/extension	−70° → 70°
Wrist (WR)	WR_z	Radial/ulnar deviation	−30° → 20°

**Table 4:** Utterance counts per command class across raw corpus, balanced dataset, training/validation partitions, Signal-Boosted training pool, and blind test set.

Command	Category	Raw	Balanced	Train (Pre-Boost)	Val	Boosted Train	Blind Test	Boost ×
SHOULDER_UP	Shoulder	~400	399	319	80	638	195	×2
SHOULDER_DOWN	Shoulder	~400	399	319	80	638	195	×2
SHOULDER_LEFT	Shoulder	~400	399	319	80	638	195	×2
SHOULDER_RIGHT	Shoulder	~400	399	319	80	638	195	×2
ELBOW_DOWN	Elbow	~400	399	319	80	638	195	×2
ELBOW_UP	Elbow	~400	399	319	80	638	195	×2
ROTATE_WRIST	Wrist	~400	399	319	80	638	195	×2
REST	Safety	~400	399	319	80	2552	195	×8
STOP	Safety	~400	399	320	79	4800	200	×15
TOTAL	—	~3600	3591	2872	719	11,818	1760	—

**Table 5:** Functionally distinct categories.

Subset ID	Adversarial Type	*N* (Expressions Shown in Docs)	Gatekeeper/SLM Stress Function	Sample Expressions (A)	Sample Expressions (B)
Category 1	Canonical Valid Commands (Positive Control)	≥40	Establish the Gatekeeper’s true-positive acceptance baseline—confirms that complete, well-formed anatomical commands are correctly passed to the SLM and correctly classified, forming the positive-control complement to Category 3’s rejections.	“raise the shoulder up”/“bend the elbow down”/“rotate the wrist”	“lower the shoulder down”/“stop”/“rest now”
Category 2	Paraphrased/Synonym Commands (Lexical Robustness)	≥40	Verify Layer 1 vocabulary tolerance and SLM generalisation to semantically equivalent phrasings of valid commands—confirms paraphrases and synonym substitutions still resolve to the intended command label.	“lift the arm upward”/“swing the shoulder to the right”/“twist the wrist a bit”	“push the elbow higher”/“ease the shoulder downward”/“cut the movement”
Category 3	Partial/Truncated Commands (Gatekeeper Traps)	≥50	Verify Gatekeeper Layer 1 keyword-count enforcement—single noun or single direction word without sufficient anatomical context to form a valid command.	“move up”/“shift left”/“shoulder”/“arm”	“go up please”/“just my elbow”/“upper arm moving”
Category 4	Out-of-Domain Chatter	≥30	Confirm Tier 5 noise rejection and Layer 1 vocabulary check block all ambient conversation with zero false-positive activations.	“what time is it”/“play some music”/“open the door”	“good morning”/“tell me a joke”/“is it raining outside”
Category 5	Conflicting Compound Commands (Priority Override Stress Tests)	≥30	Stress-test V2 Hierarchical Gatekeeper Tier 1/Tier 2 absolute priority: utterances containing both a safety keyword (STOP/REST) and one or more anatomical movement verbs in the same sentence.	“stop moving the shoulder up”/“freeze the elbow down”	“relax the shoulder down”/“halt the arm leftward”/“cancel forearm upward”

**Table 6:** Analysis results.

Number of Items Rated (*n*)	3601
Observed agreement (*P_o_*)	0.9867
Expected agreement by chance (*P_e_*)	0.1111
Cohen’s kappa (*κ*)	0.9850
Standard error of *κ*	0.00215
z statistic	458.55
95% confidence interval	[0.9808, 0.9892]
Number of disagreements	48 (1.33%)

**Table 7:** Disagreement sample expressions.

Linguistic Expression	Rater 1	Rater 2	Command After Conciliation
finished moving rest now	STOP	REST	REST
pause and rest	STOP	REST	REST
be on standby	REST	STOP	REST
arm hang down	SHOULDER_DOWN	REST	SHOULDER_DOWN
arm lay down	SHOULDER_DOWN	REST	SHOULDER_DOWN
go to break position	STOP	REST	REST
arm fully down	SHOULDER_DOWN	REST	SHOULDER_DOWN
start forearm lift	REST	ELBOW_UP	ELBOW_UP
arm done	STOP	REST	STOP
arm laid down	SHOULDER_DOWN	REST	SHOULDER_DOWN
movement done rest	STOP	REST	REST
Hold and Rest	STOP	REST	REST
arm down position	SHOULDER_DOWN	REST	SHOULDER_DOWN

**Table 8:** Per-class precision, recall, F1-score, and support against the lexical-oracle proxy ground truth (*N* = 1847).

Command Class	Support (n)	Precision	Recall	F1-Score	Notes
SHOULDER_UP	723	1.0000	1.0000	1.0000	—
SHOULDER_DOWN	102	1.0000	1.0000	1.0000	—
SHOULDER_LEFT	71	1.0000	1.0000	1.0000	—
SHOULDER_RIGHT	14	1.0000	1.0000	1.0000	—
ELBOW_UP	651	0.9985	1.0000	0.9992	1 FN (→ELBOW_DOWN)
ELBOW_DOWN	181	1.0000	0.9945	0.9972	1 FP (from ELBOW_UP)
ROTATE_WRIST	105	1.0000	1.0000	1.0000	—
Macro-average	1847	0.9998	0.9992	0.9995	—

**Table 9:** Study metric inventory: results against the seven technical evaluation requirements.

Metric	Target/Definition	Result	Status
ASR Word Error Rate	On in-domain utterances; per-language.	FPRR proxy: 76.73% (SD 10.76, range 60.43–94.74%). English-only cohort.	Proxy
Intent Classification Accuracy	Top-1 across 9 classes; per-class P/R/F1. Target ≥ 90%.	99.95% top-1 (1847 oracle-resolvable events); Macro-F1 = 0.9995.	Met
Confusion Matrix	Identify UP/DOWN, LEFT/RIGHT confusion pairs.	1 error in 1847 (ELBOW_UP → ELBOW_DOWN); no directional-pair confusion.	Met
Gatekeeper Veto—Valid	Approach 0% (false rejections).	2.02% (38/1885 oracle-resolvable); voice-level 22.53% is ASR-driven, not Gatekeeper miscalibration.	Met
Gatekeeper Veto—Adversarial	Approach 100% (true rejections).	100% (366/366) on naturally occurring off-domain speech; dedicated adversarial corpus outstanding.	Proxy
End-to-End Latency	Median and p95, end-of-speech to command emission.	Median = 2.50 s (SD = 0). p95 not computable—per-trial timestamps required.	Partial
Baseline Comparisons	(i) Keyword spotter; (ii) LLM w/o Gatekeeper; (iii) LLM w/Gatekeeper.	(i) 3.9% vs. 68.2% SLM pipeline—17.4× improvement. (ii,iii) require external API experimentation.	(i) Met; (ii,iii) Outstanding

**Table 10:** Feature-by-feature positioning of the present work.

Feature	VoicePilot [4]	BTLA [7]	RoboGuard [8]	Modular Guardrails [9]	Present Work
Language-understanding mechanism	GPT-3.5-turbo, cloud API, prompt-only	GPT-3.5-turbo, cloud API, prompt-only	Root-of-trust LLM (safety framework—not a full NL interface)	Modular LLM-as-judge components (safety framework)	Custom DistilBERT, 44 M params, on-device, fine-tuned 30 epochs
ASR handling	Whisper, cloud API	Whisper, cloud	Out of scope	Out of scope	Distil-Whisper distil-small.en, on-device, fully offline, FP16
Upstream input repair	✗	✗	✗	✗	✓ Phonetic Interceptor (anatomical-term sanitisation)
Safety-enforcement architecture	Predefined trajectories + LLM output post-processing	Single-tier STOP bypass + LLM validation	2-stage: root-of-trust LLM + temporal-logic enforcement (LLM in safety path)	Modular monitoring + intervention layers (LLM-as-judge components in safety path)	3-layer deterministic Gatekeeper: Semantic Validation, Kinematic Feasibility, Safety Constraints—no probabilistic component in safety path
Command vocabulary structure	Free-form (Python code generation)	6 predefined manipulation skills, JSON schema	N/A	N/A	9 atomic commands; structured schema { intent, magnitude, duration, confidence }
Empirical evaluation	*N* = 11 older adults; SUS = 73.0 (SD 18.6); feeding task	*N* = 10 graduate students; +76.1% coverage vs. SPB; NASA-TLX ↓ 38–52%; 85% rated “natural”	Theoretical/architectural (no user study)	Theoretical/architectural (no user study)	*N* = 14 log-derived: 99.95% classification acc., 2.02% false-rejection, 100% adversarial rejection, 17.4× vs. keyword spotter
Deployment surface	Cloud API dependency	Cloud API dependency	Framework (implementation-dependent)	Framework (implementation-dependent)	On-device (offline capable); 21.69 ms mean inference latency

## Data Availability

The training corpus, fine-tuned model weights, source code of the DAS3 Neuro-Voice Controller (AI Vocal front-end, SLM classifier, Phonetic Interceptor, and Hierarchical Gatekeeper), OpenSim DAS3 integration layer, and anonymised interaction logs supporting the analyses reported in Section 5 and Section 6 are openly available at: https://github.com/monicaleba/DAS3-Neuro-Voice-Controller (accessed on 26 August 2026). Participant identifiers are anonymised as U01–U14; no personally identifiable information is included in the released data.
